# Dietary Sources and Bioactivities of Melatonin

**DOI:** 10.3390/nu9040367

**Published:** 2017-04-07

**Authors:** Xiao Meng, Ya Li, Sha Li, Yue Zhou, Ren-You Gan, Dong-Ping Xu, Hua-Bin Li

**Affiliations:** 1 Guangdong Provincial Key Laboratory of Food, Nutrition and Health, Department of Nutrition, School of Public Health, Sun Yat-sen University, Guangzhou 510080, China; mengx7@mail2.sysu.edu.cn (X.M.); liya28@mail2.sysu.edu.cn (Y.L.); zhouyue3@mail2.sysu.edu.cn (Y.Z.); xudp@mail2.sysu.edu.cn (D.-P.X.); 2 School of Chinese Medicine, Li Ka Shing Faculty of Medicine, The University of Hong Kong, Hong Kong 999077, China; 3 School of Biological Sciences, The University of Hong Kong, Hong Kong 999077, China; ganry@connect.hku.hk; 4 South China Sea Bioresource Exploitation and Utilization Collaborative Innovation Center, Sun Yat-sen University, Guangzhou 510006, China

**Keywords:** melatonin, food, bioactivity, antioxidant, anticancer, mechanisms of action

## Abstract

Insomnia is a serious worldwide health threat, affecting nearly one third of the general population. Melatonin has been reported to improve sleep efficiency and it was found that eating melatonin-rich foods could assist sleep. During the last decades, melatonin has been widely identified and qualified in various foods from fungi to animals and plants. Eggs and fish are higher melatonin-containing food groups in animal foods, whereas in plant foods, nuts are with the highest content of melatonin. Some kinds of mushrooms, cereals and germinated legumes or seeds are also good dietary sources of melatonin. It has been proved that the melatonin concentration in human serum could significantly increase after the consumption of melatonin containing food. Furthermore, studies show that melatonin exhibits many bioactivities, such as antioxidant activity, anti-inflammatory characteristics, boosting immunity, anticancer activity, cardiovascular protection, anti-diabetic, anti-obese, neuroprotective and anti-aging activity. This review summaries the dietary sources and bioactivities of melatonin, with special attention paid to the mechanisms of action.

## 1. Introduction

Melatonin, *N*-acetyl-5-methoxy tryptamine, was first isolated from bovine pineal gland [1]. As the biological roles of melatonin were widely studied, the recognized therapeutical effects and the health benefits of melatonin could cover a broad range. Melatonin could regulate human physiological rhythm, alleviate related disorders like jet lag [2] and insomnia [3], scavenge free radical species [4], enhance the immune system [5], show anti-aging [6] and anti-inflammatory effects [7] and perform anticancer activities [8]. Moreover, melatonin could also exhibit neuroprotective effects [9], facilitate the control of chronic diseases, such as cardiovascular diseases [10], diabetes [11] and obesity [12]. In addition, melatonin could even regulate the mood [13], sexual maturation [14] and body temperature [15]. Recent research revealed the promising therapeutical application of melatonin in periodontology [16]. 

**Figure 1:**
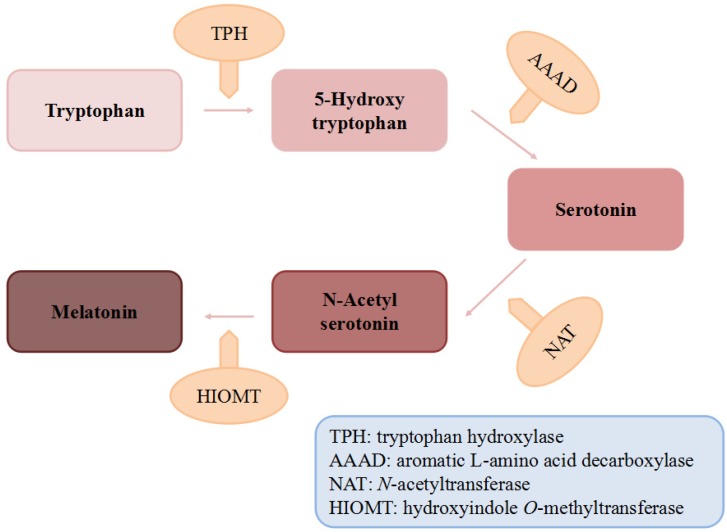
The biosynthesis of melatonin.

After being long considered as a hormone exclusively produced in the pineal gland of animals (Figure 1), melatonin has been identified in plants [17], insects [18], fungi [19] and bacteria [20]. Given the potent health effects of melatonin, many foods have been tested in the past decades and melatonin was identified and quantified in both animal foods and edible plants [21,22]. Huge differences of melatonin concentrations were reported among various food species and/or organs, ranging from pg/g to mg/g [22,23]. Additionally, it was well documented that the consumption of melatonin-rich foods may induce the potential health impacts by significantly increasing the serum melatonin concentration and antioxidant capacity in human beings [24]. Therefore, those foods containing melatonin are now popular and regarded as promising nutraceuticals [25,26,27]. 

This review outlines the dietary sources, summarizes the bioactivities of melatonin, and special attention was paid to the mechanisms of action. A search of PubMed and Web of Science was conducted, and the related peer-reviewed articles published in English within 10 years were included because of the huge number of studies. 

## 2. Dietary Sources of Melatonin

Melatonin exists widely in many kinds of food stuffs (Table 1). However, the content of melatonin in foods exhibits huge differences from species to species. Much higher melatonin was observed in nuts and medical herbs [28,29]. For the same species, it varies in different cultivars [30]. A study qualified the melatonin contents in 58 cultivars of corns, which ranged 10–2034 ng/g dry weight (DW) [31]. Additionally, in both animal foods and plant foods, melatonin could distribute unevenly in one individual animal or plant because of the different biophysical dynamic features in organs [22,32]. In edible plants, fruits generally present the lowest melatonin content, while the seeds and leaves have the highest one [33,34]. Furthermore, the melatonin concentration in the plant food products is also associated with the environment, in which the plants are cultured, including the temperature, sunlight exposing duration, ripening process, agrochemical treatment etc. [35,36]. Therefore, it is challenging and promising to clarify the factors influencing melatonin concentration in foods so as to select the effective approaches, such as germination of seeds, to increase the melatonin content in foods. 

### 2.1. Animal Foods

In animal foods, melatonin concentrations were found higher in eggs and fish than those in meat [22]. Melatonin was detected in breast milk of human beings and also in the milk provided by other animals [22,37]. Similar as the fluctuation in plasma, i.e., relatively low during daytime and relatively high at night, melatonin levels in milk also showed a circadian rhythm, indicating that night milking might increase the benefits from milk as the melatonin concentration was approximately ten times compared to that in the daytime [38]. Furthermore, melatonin was found in colostrum with the comparable concentration as it in plasma [39], which could benefit the newborns that lack the established rhythmic excretion of melatonin in the first couple of weeks in their lives [40]. Neither the artificial formulas nor the fermented milk drink were tested with detectable melatonin [41,42]. 

### 2.2. Plant Foods

#### 2.2.1. Cereals

Cereals are largely consumed all around the world, in which melatonin contents were also investigated. Wang et al. studied melatonin contents in 58 cultivars of corns and 25 of rice, finding huge variety due to genotypes: 0–2034 ng/g (mean: 96.5 ng/g) for corns and 0–264 ng/g (mean: 16 ng/g) for rice [31]. Additionally, they also discovered the pigmented rice contained higher melatonin contents, which was consistent with the findings from Setyaningsih et al. [17]. Moreover, Setyaningsih et al. reported that the level of melatonin in nonglutinous black rice was almost twice as that in the glutinous type and the melatonin concentration was 1/3 less in polished rice compared to that in the whole ones. For other cereals such as wheat, barley and oats, melatonin was found relatively high, e.g., 124.7 ± 14.9 ng/g fresh weight (FW) in wheat, 82.3 ± 6.0 ng/g FW in barley and 90.6 ± 7.7 ng/g FW in oats, respectively [34]. In terms of bread, crumb was found with higher melatonin levels than the crust [20].

#### 2.2.2. Fruits

Generally, melatonin was found in many commonly consumed fruits. Grapes [43], cherries [30] and strawberries [44] were the most popular ones in the investigation of melatonin contents, which also showed the difference between cultivars. The highest melatonin in three kinds of fruits was reported in the range of 8.9–158.9 ng/g DW in the skin of grapes (*Vitis vinifera* L. cv. Malbec) [45], 13.46 ± 1.10 ng/g FW in tart cherries (*Prunus cerasus* L. cv. Balaton) [46], and 11.26 ± 0.13 ng/g FW in strawberry (*Fragaria ananassa* L. cv. Festival) [44]. Other fruits contained melatonin at a relatively low level.

#### 2.2.3. Vegetables

Melatonin exists in lots of common vegetables, although it remains undetectable in potatoes and very low in beetroots [47,48]. Tomatoes and peppers were the most studied vegetables, and they showed relatively high melatonin concentrations in the vegetable group, i.e., 11.9 ng/g FW or 93.4 ng/g DW in pepper (*Capsicum annuum* L. cv. F26) and 14.77 ng/g FW or 249.98 ng/g DW in tomato (*Solanum lycopersicum* L. cv. Optima) [49], and 23.87 ± 2.02 ng/g FW in tomato (*Lycopersicon esculentum* cv. Bonda) [44]. 

Mushrooms also contain melatonin, e.g., 12,900 ± 770 ng/g DW in Basidiomycota (*Lactarius deliciosus*), 6800 ± 60 ng/g DW in Basidiomycota (*Boletus edulis*) [42], and 4300–6400 ng/g DW in Agaricus bisporus [50].

#### 2.2.4. Legumes and Seeds (Raw and Germinated)

Melatonin occurred in many legumes and seeds, and in some particular seeds, such as white and black mustard seeds, melatonin was as high as 189 ng/g DW and 129 ng/g DW, respectively [51]. Moreover, the germination process of legumes and seeds was proved to be able to increase the melatonin levels significantly [52,53]. For example, in germinated soybean seeds, the melatonin level reached a peak of 1.89 ± 0.11 ng/g DW during germination, which was 400% increase than that in raw soybean seeds [52]. Similarly, melatonin was observed more than 11 folds in the germinated mung bean seeds as it was in the raw ones [53]. 

#### 2.2.5. Nuts

Melatonin was found in various nuts, and pistachio (*Pistacia vera* L.) was reported with the highest content (233,000 ng/g DW) to date [28]. 

#### 2.2.6. Juices and Beverages

The popular alcoholic drinks like beer [42,54] and wine [55,56], were tested for melatonin, with 0.09 ± 0.01 ng/mL in beer [42] and up to 129.5 ± 3.5 ng/mL in wine [42,54]. Besides the melatonin originated from the main ingredients, it was also considered to be synthesized during the fermentation process due to the yeast growth [54,57].

As for the other drinks, coffee usually contains high concentration of melatonin because coffee beans contained melatonin at a very high level and even much higher in roasted beans [58]. Melatonin also existed in juices [43], cacao [42] and balsamic vinegars [59]. However, it was not found in concentrates [60] or in tea [42], including green tea and black tea. 

#### 2.2.7. Medical Herbs

Many medical herbs were analyzed and some of them contained melatonin at a level of more than 1000 ng/g DW [61,62]. In Huang-qin (*Scutellaria biacalensis*) melatonin was significantly high as 7110 ng/g DW, and in St. John’s Wort (*Hypericum perforatum*) melatonin concentration was 4490 ng/g DW in flowers and 1750 ng/g DW in leaves [29]. Besides, 108 Chinese medical herbs were quantified, and melatonin was more than 10 ng/g DW in 64 tested herbs [61]. 

#### 2.2.8. Edible Oils

Melatonin ranged 0.03–0.29 ng/g in the tested edible oils, among which refined linseed presented 0.29 ng/g and virgin soybean 0.19 ng/g [63,64]. De la Puerta et al. also demonstrated that generally the melatonin levels in the refined olive and sunflower oil were about half of those in the extra virgin olive oil [63].

#### 2.2.9. Yeast

Melatonin was found ranging 2.2 ± 0.14 ng/g in yeast, particularly *Saccharomyces cerevisae*, which is widely used in bread baking and alcohol industry [22].

Collectively, melatonin has been found in lots of foods. In animal foods, eggs and fish are relatively rich in melatonin, whereas in plant foods, nuts contains the highest melatonin contents, and some cereals and germinated legumes or seeds are also with high contents of melatonin. Mushrooms are also high-melatonin foods. However, it should be pointed out that the data on content of melatonin in foods in Table 1 were obtained by different analytical methods. Thus, quality of these data depended on the pretreatment method of sample and the analytical method itself. For example, in terms of radioimmunoassay (RIA) and enzyme-linked immunoabsorbent assay (ELISA), melatonin content could be over-estimated as some other components in the foods might also react with the related antibodies and enzymes, resulting in the lower accuracy. In addition, high-performance liquid chromatograph with ultraviolet detector (HPLC-UV) has low sensitivity and selectivity. Although high-performance liquid chromatograph with fluorescence detector (HPLC-FD) has high sensitivity, its selectivity is also low. Thus, melatonin content could be over-estimated by HPLC-UV or HPLC-FD method. Generally, gas chromatography-mass spectrometry (GC-MS) and high-performance liquid chromatography-mass spectrometry (HPLC-MS) have high resolution and sensitivity. Therefore, the data obtained by GC-MS or HPLC-MS are more accurate than those obtained by other methods [65].

### 2.3. Other Issues about Melatonin Intake

#### 2.3.1. The Alternation of Endogenous Melatonin along Life Cycle

It has been well-documented that the secretion and/or serum levels of melatonin alter through a human lifetime [66,67,68]. Specifically, the serum levels of endogenous melatonin during the day and at night were measured in 367 volunteers (210 males and 157 females), aged 3 days to 90 years [67]. The results of this study demonstrated that nighttime serum melatonin concentration was low during the first 6 months of life [27.3 ± 5.4 (±standard error, SE) pg/mL], then reached a peak (329.5 ± 42.0 pg/mL) at 1–3 years of age, followed by a huge drop to 62.5 ± 9.0 pg/mL in the group of aged 15–20 years, probably due to the body size expansion during childhood and puberty without an increase in the rate of secretion. Then an additional decline followed in adulthood until 70–90 year of age (29.2 ± 6.1 pg/mL), attributing to a degenerative pineal gland associated with aging. Overall, the alternations of nocturnal serum melatonin during lifetime showed a fragmentary and inconsistent manner. Meanwhile, daytime serum melatonin levels were low and not age-related.

#### 2.3.2. Bioavailability of Exogenous Melatonin

Melatonin has been known and used to assist sleep disorders [8] or jet lag [69] as a dietary supplement or as a drug, and recently it has also been reported to synergize other drugs as an adjuvant to improve their efficiency or to attenuate their side effects [70]. Herein, the bioavailability of melatonin has been studied by some researchers. In a study in vivo among 12 healthy volunteers, melatonin was administrated intravenously (i.v.) by 2 mg and orally by 2 and 4 mg, and in neither of these two phases the difference in serum half-life was found [71]. This study revealed the poor absolute bioavailability of oral melatonin tablets in both dosages, which was approximately 15%, possibly because of the poor oral absorption, large first-pass metabolism, or combined. Moreover, another study was conducted with 12 young healthy volunteers (6 males, 6 females), with 250 μg oral solution of D_7_ melatonin, a molecule in which seven hydrogen atoms are replaced by seven deuterium atoms. It was reported that the absolute bioavailability of melatonin ranged from 1% to 37% and higher in women (16.8% ± 12.7%) than in men (8.6% ± 3.9%). It was also observed that the apparent terminal half-life values were 36 ± 2 and 41 ± 10 min for males and females respectively, indicating the fast-release property of melatonin [72]. Besides, 7 healthy male volunteers (ages 31.1 ± 1.1) were given an ingestion of 3 mg melatonin, and a marked increase in serum melatonin (3561 ± 1201 pg/mL) was found within 20 min, followed by a gradual decrease, but the level still remained higher than the basal level at 240 min after the ingestion [73]. 

#### 2.3.3. Benefits of Consuming Melatonin-Containing Foods

Since the secretion of endogenous melatonin decreases after childhood, increasing dietary consumption could be a good option. The studies showed that intake of the food rich in melatonin may gain health impacts by increasing circulating melatonin [24,74,75,76,77]. For instance, when the rats were fed with walnuts (*Juglans regia* L.) containing melatonin concentrations of 3.5 ± 1.0 ng/g, the increased blood melatonin concentrations and total antioxidant capacity of blood were observed, indicating walnuts could provide beneficial effects as a good food source of melatonin [78]. Additionally, in a study conducted with young, middle-aged and elderly participants (20 ± 10 year-old, 45 ± 10 year-old and 75 ± 10 year-old, respectively), the total antioxidant capacity were reported to significantly increase in the three groups of individuals after the intake of the experimental juice of grape (*Vitis vinifera* cv. Tempranillo), 200 mL twice a day (as the lunch and dinner desserts) for 5 days [76] as well as the urinary 6-sulfatoxymelatonin, a major metabolite of melatonin commonly used as a biomarker indicating its bioavailability [79]. Moreover, it was found in vivo in 12 healthy male volunteers that the consumption of tropical fruit (banana) or fruit juices (orange and pineapple) significantly increased the serum melatonin concentration and the highest value was observed at 120 min after intake, i.e., compared with before consumption, pineapple with 146 pg/mL versus 48 pg/mL (*p* = 0.002), orange with 151 pg/mL versus 40 pg/mL (*p* = 0.005), and banana with 140 pg/mL versus 32 pg/mL (*p* = 0.008), respectively. Besides, the antioxidant capacity in the serum also markedly increased, suggested by the significant increases in two indicators, i.e., ferric reducing antioxidant power (FRAP) assay and oxygen radical antioxidant capacity (ORAC) [24]. Furthermore, as the germination of legumes significantly increased the melatonin content, it was reported that in Sprague-Dawley rats, the melatonin concentrations in plasma increased by 16% (*p* < 0.05) after the administration of kidney bean sprout extract via gavage, which correspondingly led to the increase of urinary 6-sulfatoxymelatonin content (*p* < 0.01), antioxidant capacities did not show significant variation though [80]. 

#### 2.3.4. Guidance on Regulating Dietary Supplement of Melatonin

Dietary supplement of melatonin could be another option to recompense the physiologically declined activity of pineal gland. Different authorities have published their guidance on regulating the dietary supplements including melatonin. For instance, European Food Safety Authority published the Scientific Opinion on the substantiation of a health claim related to melatonin and reduction of sleep onset latency (ID 1698, 1780, 4080) pursuant to Article 13(1) of Regulation (EC) No. 1924/20061 in 2010. The Panel on Dietetic Products, Nutrition and Allergies considers that “melatonin is sufficiently characterized” and “reduction of sleep onset latency might be a beneficial physiological effect”. In addition, it was also concluded that “a cause and effect relationship has been established between the consumption of melatonin” and “The target population is assumed to be the general population”. Moreover, the dose was also suggested as “In order to obtain the claimed effect, 1 mg of melatonin should be consumed close to bedtime” [81]. While according to the latest updated data (on 22 March 2013) from Food Standards Australia New Zealand, melatonin was listed in the original table 3 on their official website [82], which presents the foods or properties of food from food-health relationships derived from 20 EU approved health claims that will not be added to Standard 1.2.7 and the rationale for exclusion “The claim refers to a dietary supplement.” Additionally, in each of the databases, named “AUSNUT 2011-13 Australian Health Survey (AHS) Dietary Supplement Details” and “AUSNUT 2011-13 Australian National Nutrition Physical Activity Survey (NNPAS) Dietary Supplement Nutrient Database”, only one dietary supplement of melatonin was listed with the same information, i.e., dietary supplement name: Nature’s Care Melatonin; ID 122196; 3 mg, Tablet, uncoated; classification code 33505 and classification sub-group, other supplements [82]. 

## 3. Bioactivities of Melatonin

Melatonin has been well known as a potent antioxidant, anti-inflammatory factor and immune modulator. It also contributes to regulating the circadian system [100], possesses tumor inhibitory properties [7], and shows its benefits on cardiovascular functions [101], blood pressure [101], and lipid and glucose metabolism [102] (Table 2). 

### 3.1. Antioxidant Activities

Reactive oxygen species (ROS) and reactive nitrogen species (RNS) play an important role in a variety of physiological processes, such as regulating vascular tone, controlling ventilation, producing erythropoietin and transducing signals [103,104]. Importantly, at a desirable level they can promote cell survival, proliferation and differentiation [105]. However, excessive free radicals could result in DNA and RNA damage, protein denaturation, lipid peroxidation, leading to cell apoptosis or necrosis. Consequently, a series of health problems occur, including aging [106], inflammation [107], cancer [108], chronic metabolic disease [109,110], neurodegenerative disorders [9] and sepsis [111]. Many natural products have shown antioxidant activities and free radical scavenging capabilities [112,113,114,115,116], which could be used to prevent and treat the diseases induced by oxidative stress [117,118,119,120,121]. 

As a powerful endogenous radical scavenger, melatonin can directly remove the excessive free radicals. In addition, melatonin at 10 mg/kg was found to increase the efficiency of electron transport chain in mitochondria in old mice to lower electron leakage and reduce free radical generation [122]. Therefore, melatonin is essential to keep a stable physiological status in human body. Moreover, it could effectively play a role by modulating and acting synergistically with other reducing molecules like reductases [123] and some non-enzyme reductants [124], all of which work together to maintain normal homeostasis. 

**Figure 2:**
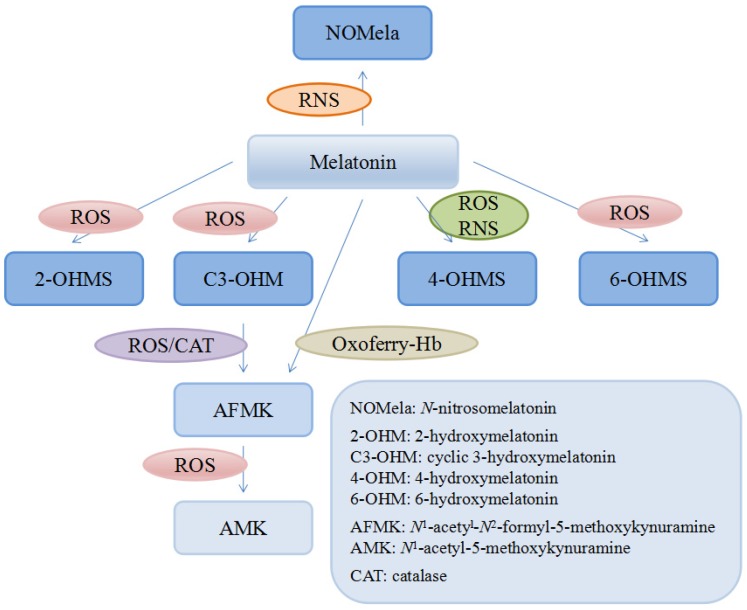
Melatonin and its metabolites.

There are many kinds of reductants in human body like glutathione, NADH and vitamin C or E, compared to which melatonin was documented as a stronger antioxidant in eliminating some free radicals both in vitro and in vivo [123,125]. For instance, it was reported that in vitro the ability of melatonin to scavenge the hydroxyl radical (∙OH) was much higher compared with that of vitamin E [123], glutathione and mannitol [126]. Another in vitro study showed that melatonin could significantly inhibit the vasoconstriction induced by H_2_O_2_ in the human umbilical artery in a dose-dependent manner [127]. Moreover, it was found that in rats, melatonin was several times more powerful than vitamin C and E in protecting tissues from injuries induced by oxidative stress [125,128]. In addition, quite different from the other antioxidants, the metabolites of melatonin are also actively involved in scavenging free radicals, referred as cascade, though they are the intermediates in the process of reactions against radicals [129] (Figure 2). Interestingly, some of the metabolites are even more potent than its precursor. A good example is that the capacity of 3-hydroxymelatonin (C3-OHM) to reduce hypervalent hemoglobin was higher than that of its basic form and another example was *N*^1^-acetyl-5-methoxykynuramine (AMK) showing stronger capability of scavenging ROS and preventing protein oxidation than its precursor [130,131]. Such a profile greatly increases the effectiveness of melatonin as a powerful free radical scavenger. Considering the cascade effects of the metabolites, a melatonin molecule could scavenge up to 10 ROS/RNS molecules [131].

Numerous evidence supports that melatonin is a broad-spectrum free radical scavenger [132,133,134]. In addition to ROS/RNS, many other molecules could be modulated or scavenged by melatonin and its metabolites, such as hemoglobin-derived oxoferryl radicals [135]. Furthermore, in vitro and/or in vivo, melatonin is able to chelate toxic metals such as cadmium [136], mercury [137], arsenic [138], lead [139], aluminum [140], chromium [141], which are involved in the generation of free radicals. Moreover, melatonin and its metabolites were also documented to exhibit free radical avoidance properties, by downregulating pro-oxidative enzymes like inducible nitric oxide synthase (iNOS) both in vitro (dose-dependent) and in vivo as well as inhibiting the mRNA expression of cyclo-oxygenase 1 (COX-1) and COX-2 in human breast cancer cells (MCF-7) [142,143]. 

Melatonin also stimulates the synthesis of other antioxidants. For instance, melatonin was found to induce the expression of gamma-glutamylcysteine synthetase (γ-GCS), the rate-limiting enzyme of GSH synthesis, in human vascular endothelial cells (ECV304) [124]. It was also observed that in 2 neuronal cell lines, melatonin could regulate the expression of antioxidant enzymes (AOEs) at 1 nM under normal conditions, showing the mRNA increase of superoxide dismutase (SOD), glutathione peroxidase (GPx) [144]. Moreover, in vivo and/or in vitro melatonin prevents antioxidant enzymes from oxidative stress, and increases the activities of other redox enzymes, such as catalase (CAT) [145], glutathione reductase (GSH-Rd) [146], and glucose-6-phosphate dehydrogenase (G6PD) [147]. Besides, in vitro melatonin could combine with other antioxidants including vitamin C, vitamin E, glutathione, at the concentration of 2.5–1600 μM, leading to the markedly enhanced protective effects of removing radicals synergistically [148]. 

### 3.2. Anti-Inflammatory Activities

Inflammation can commonly occur locally and systematically, caused by many external or intrinsic factors. It has been well documented that the complex interaction of oxidative stress and inflammation results in many diseases. Melatonin could not only reduce the oxidative levels as stated, but also effectively fight against inflammation via different mechanisms such as essential signaling pathways [70,149], the modulation of related genes [150] and the activation membrane receptors [151], as discussed in detail below.

#### 3.2.1. NF-κB Signaling Pathway Involved Mechanisms

Nuclear factor kappa-light-chain-enhancer of activated B cells (NF-κB) is distributed in nearly all the types of animal cells and it is well known to control DNA transcription, cytokine production and cell survival [152,153]. It is an important pro-inflammatory factor that plays a pivotal role in oxidative stress-induced inflammation. In numerous research, melatonin was found to exert anti-inflammatory activity by suppressing NF-κB signaling pathways [70,154,155,156,157]. Melatonin and its metabolites such as AFMK and AMK were able to modulate NF-κB and its downstream pro-inflammatory target genes such as iNOS [70], COX-1 [158], COX-2 [156], tumor necrosis factor α (TNF-α) [159], glia and fibrillary acidic protein (GFAP) [160], which contributed to the pathophysiology of many diseases. In vitro study, melatonin exerted cytoprotective and anti-inflammatory effects on oxidative stress-stimulated human chondrocytes by blocking the activated NF-κB as well as the phosphorylation of phosphatidyl inositol 3-kinase (PI3K)/Akt, p38, extracellular signal-regulated kinase (ERK), Jun *N*-terminal kinase (JNK), and mitogen-activated protein kinase (MAPK) in a dose- and time-dependent manner [161]. Another in vitro study showed melatonin at 1 nM also performed its anti-inflammatory functions by downregulating chemokine expression via the inhibition of NF-κB, signal transducer and activator of transcription (STAT)1/3 phosphorylation, and gamma-activated sequence (GAS)-driven transcriptional activity in lipopolysaccharide (LPS)-stimulated BV2 murine microglial cell line [162]. Additionally, the protective effect of melatonin on cigarette smoke-induced restenosis in rat carotid arteries after balloon injury was also observed as melatonin inhibited the inflammatory reaction via NF-κB signaling pathways [163]. Moreover, in mice with LPS-induced mastitis, besides the suppression of LPS-induced NF-κB activation, activating peroxisome proliferator-activated receptor gamma (PPAR-γ) was regarded as another underlying anti-inflammatory mechanism of melatonin [164]. A recent study indicated that exogenous melatonin could inhibit inhibitor of nuclear factor kappa-B kinase (IKK)/NF-κB signal transduction pathway in stimulated mast cells (RBL-2H3) to prevent inflammation and the effects were directly related with melatonin concentration used at 100 nM and 1 mM, based on which melatonin was implied to be used for the treatment of allergic inflammatory diseases [165]. 

#### 3.2.2. SIRT1 Pathway Involved Mechanisms

It was suggested that human sirtuins may function as intracellular regulatory proteins with mono-ADP-ribosyl transferase activity, and sirtuin 1 (SIRT1), also known as NAD-dependent deacetylase, was reported to improve insulin sensitivity, as it could affect the activity of both the estrogen-related receptor alpha (ERR-α) and the peroxisome proliferator-activated receptor gamma coactivator-1 alpha (PGC-1α), which are essential metabolic regulatory transcription factors [149]. Specifically, SIRT1 was able to independently enhance ERR-α and modulate the effects of PGC-1α repression of glycolytic genes [166,167]. In a rabbit model with osteoarthritis (OA), intra-articular injection of melatonin at 20 mg/kg significantly reduced cartilage degradation, which was reversed by sirtinol, indicating SIRT1 pathway was involved [161]. Additionally, both in vitro and in vivo studies showed that melatonin reduced LPS-induced oxidative stress damage, acute neuroinflammation, and apoptotic neurodegeneration via SIRT1/Nrf2 (nuclear factor-erythroid 2-related factor 2) signaling pathway activation [168]. 

In addition to the pathways mentioned above, the anti-inflammatory effects of melatonin were also observed as it regulated the expression of some pro-inflammatory genes [150]. Moreover, it was found that melatonin could inhibit the expression of inflammatory chemokines/cytokines, i.e., chemokine (C-X-C motif) ligand 1 (CXCL1), chemokine (C-C motif) ligand 20 (CCL20), and interleukin 6 (IL-6) that was mediated by IL-17 and enhanced by increased insulin and insulin-like growth factor 1 (IGF-1) in the prostatic tissues of obese mouse through a glycogen synthase kinase 3β (GSK3β)-dependent mechanism [169]. Another study found that melatonin at 10 μM could show its anti-inflammatory impacts time-dependently by inducing temporal up-regulation of gene expression related to ubiquitin/proteasome system (UPS) in the human malaria parasite *Plasmodium falciparum* [170]. Additionally, it was reported that melatonin (10 mg/kg, intraperitoneally, i.p.) attenuated colitis with sleep deprivation in mice by downregulating mRNA of E2F transcription factor (E2F2) and histocompatibility class II antigen A, beta 1 (H2-Aβ1), indicating its clinical potential for patients with inflammatory bowel disease, particularly those suffering from sleep disturbances [171]. Furthermore, melatonin was reported to reduce intestinal ischemia-reperfusion-induced lung injury in rats dose-dependently by activating the expression of *N*-myc downstream-regulated gene 2 (NDRG2), which was involved in cellular differentiation, development, anti-apoptosis, anti-inflammatory cytokine, and antioxidant [172]. However, it should be pointed out that those results were from different animal models. If the same animal model was used, different results might be observed. 

Besides, it has been reported that melatonin receptors were involved in its anti-inflammatory mechanism. It was found that melatonin played a role in maintaining the pro- and anti- inflammatory balance during infection by influencing leukocyte migration and apoptosis in carp possibly mediated by melatonin MT1 receptors in/on leukocytes [151]. 

### 3.3. Enhancing Immune Activities

Recently, numerous experimental evidence has shown that melatonin is involved in the interaction between the nervous, endocrine, and immune systems on the basis of the research on surgical or functional pinealectomy as well as the association between melatonin production and circadian and seasonal rhythms in the immune system [173,174,175]. In these three interactive systems, the reciprocal regulation exists. For instance, the immunological parameters such as TNF-α [176], IL-12 [177], interferon gamma (IFN-γ) [178], granulocyte colony-stimulating factor (G-CSF) and granulocyte–macrophage colony-stimulating factor (GM-CSF) [179] can affect the function of pineal gland. In addition, NF-κB could be active during pathogen invasion and melatonin synthesis could be promoted in macrophages while inhibited in pinealocytes. Therefore, immune-pineal axis has been defined as the shift in the melatonin production from pinealocytes to immune competent cells mediated by NF-κB [180,181]. Furthermore, melatonin exerted the neuroimmunomodulatory effect on the immune system via its membrane receptors, which have been identified in immune organs, tissues, bone marrow mononuclear cells (BMMNCs) and leukocytes, and even subcellular compartments [180,182]. With MT1 and MT2 receptors, melatonin was found to inhibit the production of forskolin-stimulated cyclic AMP (cAMP), cyclic GMP (cGMP) and diacylglycerol (DAG), leading to the improved immunity [183,184]. Besides, the specific nuclear melatonin receptors have been identified in Jurkat cells [185], lymphocytes [186], thymus [175] and spleen [187], which belong to the RZR/ROR subfamily of nuclear receptors.

It was observed that melatonin could reverse the weight loss of thymuses [175] and spleens [188] in different pinealectomized animal models and melatonin could increase tonsillar size [189], indicating the protective effects of melatonin on the immune organs. Besides, melatonin and its metabolites like AFMK were found to improve the proliferation, increase the activity and inhibit apoptosis of immune competent cells such as monocyte [190], natural killer (NK) cells [191] and neutrophils [192]. Melatonin could act on the membrane receptors MT1 and MT2 and increase the sensitivity of the immune cells to some cytokines such as TNF-α and IFN-γ in vivo at the dose of 10 mg/kg orally administrated [193]. Furthermore, Ghosh et al. found that melatonin could restored the suppressed immunity of T-cell culture in vitro, indicating melatonin might be valuable in regulating immunity via the functional interactions with gonadal steroid by developing some hormonal microcircuit (gonadal steroid and melatonin) in lymphatic organs [194]. Moreover, melatonin was able to modulate immune mediator production, e.g., increased IL-2, IFN-γ and IL-6 in monocytes [195] in cultured human mononuclear cells, decreased IL-8 and TNF-α in neutrophils [192], decreased IL-1β, IL-6, IL-8, IL-10 and TNF-α in macrophages in RAW264.7 cells [196]. This profile is of great importance as some cytokines have been shown to interact with immune cells and promote their growth, differentiation, activation, and survival. Besides the endocrine actions from the pineal melatonin, the melatonin synthesized in immune system could exhibit direct immunomodulatory effects by means of nonendocrine actions, including intra-, auto-, and/or paracrine actions via its membrane and/or nuclear receptors [197], which were crucial for human lymphocytes to generate an accurate response by modulating the IL-2/IL-2R system [198]. 

Melatonin was reported to regulate the ROS production in the essential immune cells such as monocytes [199] and neutrophils [200]. Moreover, melatonin was found to augment the general immunity by attenuating oxidative load associated with age in hamsters by 25 μg/100 g body weight for 30 days [201]. It was reported that melatonin could alleviate oxidative damage and suppress the immune status induced by stressful factors via its membrane receptor expression MT1 and MT2 in wild birds [202]. Due to its anti-inflammatory properties, melatonin could suppress systemic innate immune activation during sepsis in mice both in vivo and in vitro by blocking the NF-κB/NOD-like receptor P3 (NLRP3) connection through a sirtuin1-dependent pathway [154].

Collectively, it has been well documented that melatonin played a fundamental role in resisting harmful invasion and enhancing immunity as a member of the complex neuro–endocrine–immunological system. 

### 3.4. Improving Circadian Rhythm and Sleep

It was estimated that about one-third of the general population is suffering from sleep disorders, mainly insomnia, and there is an increasing trend because of the more stressful working conditions and the progressive aging of society [203]. Circadian rhythms are common in nature and have been widely observed in cyanobacteria, fungi, plants, and animals [204]. Disturbance of circadian rhythms can cause many diseases like cancers [205], metabolic syndromes [206], reproductive diseases [207] etc. Disruption of circadian rhythms and sleep disorders are prevalent, which can do harm to patients’ overall health, worsen existing comorbidities and result in impaired quality of life, whereas melatonin could modulate circadian rhythm and improve sleep disorders [100,208,209,210]. 

In mammals, most of the physiological processes and behaviors are regulated by a network of circadian clocks. The circadian system consists of a central rhythm generator, the suprachiasmatic nucleus (SCN), and several peripheral oscillators [211]. Besides, the central clock can control the production of melatonin, which could modify the peripheral clocks and inversely alter the expression of circadian clock genes. Johnston et al. [212] found that rising melatonin levels could reset circadian rhythms in the mammalian pars tuberalis. Melatonin has long been known to help prevent and treat jet lag, a typical example of disrupted circadian rhythms often caused by travelling [2].

The animal models of melatonin-proficient (C3H) and melatonin-deficient (C57BL) mice are frequently used to study the role of melatonin on circadian rhythms. In a study, research on three clock gene proteins PER1, BMAL1 and CRY2 in the murine adrenal cortex and medulla was conducted and the results showed that in C3H mice, PER1 and CRY2 maximized in the middle of the light phase, whereas BMAL1 reached its peak in the dark phase and these three clock gene proteins levels displayed day/night variation in both the adrenal cortex and medulla. Similar patterns were revealed in the adrenal medulla of C57BL mice, but in the adrenal cortex of C57BL mice, clock gene protein levels were consistently lower than in C3H mice and did not change with time [211]. In another study, the modulatory effects on clock gene expression of melatonin was investigated in the retina of those two groups of mice, and the results demonstrated that melatonin functioned via post-transcriptional mechanisms and also played a role in rhythmic regulation of phosphorylated cAMP response element-binding protein (pCREB) levels in the mammalian retina [213]. 

As an external trigger of melatonin production, light, blue-enriched light in particular, was observed to significantly suppress the nocturnal increase in endogenous melatonin levels in human, indicating that a clock gene polymorphism could modulate light sensitivity in humans, especially in the individuals, who are homozygous for the PER3 5/5 allele [214]. Additionally, it was observed that melatonin activated Npas4, which drove the clock gene CRY1 responses to melatonin in vivo [215]. Furthermore, Bracci et al. [216] reported that alterations in peripheral clock gene expression, i.e., a significantly higher expression of BMAL1, CLOCK, NPAS2, PER1, PER2, and REVERBα and a lower expression of PER3, CRY1 and CRY2, were found at the beginning of the morning shift after a day off in rotating shift work nurses as well as significantly higher 17-β-estradiol levels compared to daytime nurses. Another study reported that both Period1 and BMAL1 expression increased in the hippocampus of Siberian hamsters after acute melatonin treatment of 20 μg/day, accompanied by the alterations of dendritic morphology, indicating melatonin could act as a signal to coordinate the circadian rhythm in neuronal remodeling [217]. Additionally, melatonin was found to adjust the expression pattern of clock genes (per1, per2, bmal1 and clock) in the SCN mainly by increasing amplitude in their expressional rhythms without inducing robust phase shifts in them. In addition, melatonin could alter the expression of genes of serotonergic neurotransmission (tph2, sert, vmat2 and 5ht1a) in the dorsal raphe and serotonin contents in the amygdala, and improve the depression-like behavior in C57BL/6J mice with seasonal affective disorder [218]. 

Melatonin has been known as the ‘hormone of darkness’ as its synthesis and secretion are controlled by light/dark cycles, i.e., its production decreases during daytime and increases at night. The decreased nocturnal plasma melatonin levels were found in patients with long-lasting insomniac complaints, indicating circadian rhythm dysfunctions [219]. Additionally, it was reported that sleep parameters were positively correlated with melatonin secretion in patients with insomnia in coronary care unit [220]. Furthermore, melatonin was found to regulate sleep by activating receptor MT1, MT2 and enhancing the excitability of medial lateral habenula (MLHb) neurons in rats [221].

Besides, a number of studies have shown that melatonin and melatoninergic agents were effective in the treatment of insomnia, as it could accelerate sleep initiation, increase sleep duration and slightly alter sleep architecture [222,223]. In addition, due to the short half-life of melatonin in circulation, a prolonged-release melatonin medicine (Circadin^®^) [224], melatonin derivatives (e.g., ramelteon) [203], another melatonin agonists (e.g., agomelatine) [225,226] have been approved by authorities in the EU and USA to treat insomnia and resynchronize circadian rhythms.

### 3.5. Anticancer Activities

According to the World Cancer Report 2014 from WHO, cancers are among the leading causes of morbidity and mortality all around the world, with approximately 14 million new cases and 8.2 million cancer related deaths in 2012 and the number of new cases is expected to rise by about 70% over the next 2 decades [227]. Many foods were found to contain natural components with strong anticancer activities, indicating the potential for the prevention and treatment of different cancers [7,228,229,230,231].

Melatonin has been proved to be highly involved in the etiology, development, metabolism, metastasis, and therapy of different subsets of tumors. The mechanisms include inhibiting tumor cell growth and proliferation [232], modulating the metabolism of tumor cells [233], promoting apoptosis [234], exerting antimetastatic and antiangiogenic effects [235], enhancing the sensitivities to the anticancer drugs, attenuating the side effects of radio- and chemotherapies [157]. In addition, mechanisms of melatonin impacts on cancers comprise the actions of melatonin receptors MT1 and MT2, the regulation of relevant genes expression and the modulation of some signaling pathways [236,237].

#### 3.5.1. Effects on Tumor Cell Cycle, in Terms of Growth, Proliferation, Metabolism and Apoptosis

A study conducted by Wu et al. [232] demonstrated that both in vitro and in vivo, melatonin inhibited gastric tumor growth and peritoneal dissemination through the activation of endoplasmic reticulum (ER) stress and the inhibition of epithelial mesenchymal transition (EMT) via calpain-mediated C/enhancer-binding protein beta (EBPβ) and NF-κB cleavage. Another study demonstrated that melatonin could suppress breast cancer cell proliferation by exhibiting an anti-aromatase effect on hormonal positive breast cancer cells through the selective estrogen enzyme modulators (SEEMs) mechanism [238]. Furthermore, Hevia et al. reported that melatonin (1 mM) reduced glucose uptake and modified the expression of GLUT1 transporter in prostate cancer cells, resulting the attenuated glucose-induced tumor progression and the prolonged lifespan of tumor-bearing mice [233]. Moreover, Sohn et al. reported that the antiangiogenic properties of melatonin (1 mM) in hypoxic PC3 prostate cancer cells were mediated by enhancing the expression of miRNA3195 and miRNA374b [239]. Melatonin at pharmacological concentrations was able to significantly induce apoptosis of colorectal cancer LoVo cells in a dose-dependent manner via histone deacetylase 4 (HDAC4) nuclear import and decreasing H3 acetylation on Bcl-2 promoter, resulting in reduced Bcl-2 expression, which were mediated by inactivating Ca^2+^/calmodulin-dependent protein kinase II alpha (CaMKIIα) [240]. Besides, melatonin was found to enhance arsenic trioxide-induced apoptotic cell death at the dose of 2 mM by sustainably upregulating Redd1 expression and inhibiting mTORC1 upstream of the activated p38/JNK pathways in human breast cancer cells [234].

#### 3.5.2. Effects on Invasion and Metastasis of Tumor Cells

Melatonin (1 mM) could modulate motility and invasiveness of HepG2 cell in vitro through the upregulation of tissue inhibitor of metalloproteinases 1 (TIMP-1) and the attenuation of matrix metalloproteinase-9 (MMP-9) expression and activity by inhibiting NF-κB signaling pathway [241]. Melatonin was found to suppress the transactivation of MMP-9 and the metastasis of renal cell carcinoma (the most lethal of all urological malignant tumors with the potent metastasis potential) via the inhibition of Akt-MAPKs pathway and NF-κB DNA-binding activity [242]. Additionally, melatonin showed its oncostatic, antimetastatic and antiangiogenic effects in breast cancer both in vitro and in vivo by blocking proliferation of tumor cells and inhibiting the expression of Rho-associated kinase protein (ROCK-1), one of the regulatory and effector molecules in charge of migration/invasion, which could promote tumor growth and metastasis when its expression was increased [235].

#### 3.5.3. Therapy Adjunct in Tumor Treatment

**Figure 3:**
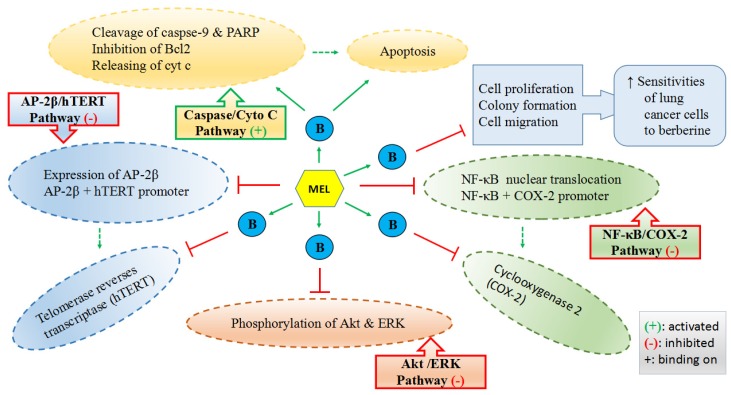
Mechanisms of melatonin enhancing the sensitivities of lung cancer cells to berberine (B).

Lu et al. reported that melatonin could enhance the antitumor activity of berberine (B in Figure 3) in lung cancer cells by activating caspase/cytochrome c (cyt c) and inhibiting activator protein 2β (AP-2β)/human telomerase reserve transcriptase (hTERT), NF-κB/COX-2 and Akt/ERK signaling pathways as well as increasing the sensitivities of lung cancer cells to berberine (Figure 3) [157]. Alonso-Gonzalez et al. pointed out that the pretreatment of 1 nM melatonin before radiation could elevate the sensitivity of human breast cancer cells to radiotherapy by reducing the active estrogens levels in cancer cells through the increased p53 expression [243]. It was also found that melatonin-based creams could significantly lower the occurrence of acute radiation dermatitis in a double-blind randomized trial [244]. It was revealed that melatonin, as a powerful antioxidant, played a protective role at 10 mg/kg in rats to alleviate the testicular dysfunction induced by chemotherapy against testicular cancer [245], which is one of the most common cancers in men of reproductive age with a steadily increasing incidence [227]. Another in vivo study reported that melatonin (25 mg/kg) could synergize the chemotherapeutic effect of 5-fluorouracil (one of the most commonly used chemotherapeutic agents to treat colon cancer) in mice with colon cancer, by promoting the activation of the caspase/poly-ADP-ribose polymerase (PARP)-dependent apoptosis pathway, inhibiting PI3K/AKT and NF-κB/iNOS signaling pathways. The results of the same study also demonstrated that melatonin significantly enhanced the 5-fluorouracil-mediated inhibition of cell growth and metastasis of colon cancer cells [70]. Additionally, a meta-analysis of randomized controlled trials reported that melatonin, as an adjuvant, could substantially reduce the side effects caused by radiochemotherapy, presenting the improved tumor remission and the increased 1-year survival [246]. 

It was also observed that MT1 and MT2 mRNA expression levels increased markedly in vitro and in samples from cancer patients, indicating that the receptor-mediated mechanisms were involved in the anticancer activities of melatonin [236,237].

### 3.6. Cardiovascular Protection

Cardiovascular diseases (CVDs) are the No. 1 cause of death worldwide, which accounted for 31% of all global deaths in 2012, i.e., an estimated 17.5 million people died from CVDs [247]. It has been found that melatonin is involved in the pathophysiological process of CVDs, such as plaque formation, atherosclerosis, and infarction. Melatonin was reported to regulate platelet physiology [248], protect the vascular endothelium [249], regulate lipid and glucose metabolism and modulate blood pressure [250], by reducing atherosclerosis, inhibiting thrombosis, which contribute to prevent CVDs or facilitate the restoration of morphology and functions of heart and vessels after injury based on reducing oxidative stress, inhibiting inflammation, decreasing apoptosis and preventing postinfarction remodeling of cardiomyocyte [251]. 

It was reported that melatonin could reduce heart rate and blood pressure, regulate the cardiac rhythm and the vascular tone via neurohumoral regulation in which the antioxidant capacity was involved [252,253]. Moreover, melatonin has been reported to protect cardiomyocytes and blood vessels against fibrosis necrosis and vasculitis in radiation-induced heart disease in rats when administrated at 50 mg/kg [254]. Furthermore, melatonin was demonstrated to dose-dependently reduce flow shear stress-induced injury in bone marrow mesenchymal stem cells (BM-MSCs) through the activation of melatonin receptors and adenosine monophosphate activated protein kinase (AMPK)/ACC (acetyl-CoA carboxylase) signaling, which could benefit the patients with valvular heart disease [255].

Melatonin was proved to increase the survival of adipose-derived mesenchymal stem cells in infarcted heart in mice at 20 mg/kg by activating SIRT1 signaling [256]. Besides, melatonin was found to significantly reduce the infarct size in myocardial ischemia-reperfusion injury (IRI) and decrease apoptosis through the activation of Janus kinase 2 (JAK2)/STAT3 signaling and PI3K/Akt signaling [257,258]. Additionally, it was reported that melatonin at 5 μM could improve the cardiac regularity in Drosophila melanogaster without affecting heart rate, possibly via a specific G-protein-coupled receptor which is encoded by the CG 4313 gene and considered to be a candidate melatonin receptor, indicating melatonin might play an essential role in cardiac pacemaking [259]. Besides, melatonin was closely related with reverse remodeling after cardiac resynchronization therapy in patients with heart failure and ventricular dyssynchrony [260]. Furthermore, it was observed that melatonin could markedly improve cardiac dysfunction, mitigate cardiac remodeling after myocardial infarction, increase the level of autophagy, attenuate apoptosis, regulate the integrity and restore the function of mitochondria through the inhibition of mammalian Ste20-like kinase 1 (Mst1) and the promotion of SIRT1, indicating the Mst1/SIRT1 signaling was involved [261]. 

Melatonin has also been found to modulate blood pressure and protect the organs and tissues damaged by hypertension. As reported by WHO, hypertension is one of the leading risk factors for CVDs and it caused 9.4 million deaths in 2010. The worldwide prevalence of hypertension in adults aged 18 years and over was around 22% in 2014 [262]. Melatonin could reduce blood pressure, pulsatility index in the internal carotid artery and catecholamines, indicating melatonin was involved in the renin-angiotensin-aldosterone system, which plays an essential role in blood pressure regulation [250]. In addition, it has been demonstrated that melatonin could regulate blood pressure both experimentally and clinically [263,264]. Hung et al. reported that melatonin was able to improve endothelial dysfunction, suppress vascular inflammation, and ameliorate systemic hypertension in rats on the basis of antioxidant and anti-inflammatory properties [265]. In addition, it was observed that melatonin could mitigate renal injury induced by hypertension by reducing oxidative stress [266,267]. It was demonstrated that melatonin could attenuate intracranial hypertension by reducing cerebral edema via the anti-inflammatory mechanism [268]. Besides, melatonin exerted the regulatory effects on blood pressure due to the interaction with both cardiovascular system and the central nerve system (CNS), by restoring the balance between the sympathetic and parasympathetic vegetative system in favor with the latter [267]. Furthermore, melatonin could even reduce fetal blood pressure in ovine fetus via the membrane receptors MT1 and MT2, possibly by releasing endothelin [269].

Several studies showed that melatonin could reduce the levels of blood lipid, another risk factor for CVDs. Dyslipidemia is commonly accompanied with other disorders related to metabolic syndromes. Melatonin has been observed to effectively improve dysplipidemia by reducing LDL cholesterol levels, total cholesterol and triglycerides, but increase HDL-C, glucose tolerance and antioxidant potency due to its potent antioxidant effects [270,271,272].

### 3.7. Anti-Diabetic Activities

According to the WHO, an estimated 1.5 million deaths were directly caused by diabetes and another 2.2 million deaths were attributable to high blood glucose in 2012. Diabetes prevalence has been rising more rapidly, and there were 422 million diabetic people in 2014. Diabetes can cause severe complications leading to disability and death, and diabetes will be the 7th leading cause of death in 2030, as estimated [273]. It has been investigated that melatonin was able to prevent the complications by attenuating glucotoxicity to the organs and tissues mainly because of its antioxidant, anti-inflammatory and antiapoptotic effects.

Melatonin was found to increase the inhibited activity of catalase in rat liver cells and restore the dysfunctional mitochondria related to diabetes at the dose of 10 mg/kg, indicating melatonin could be a beneficial option to treat diabetes [274]. It was also shown that melatonin could improve dysglycemia in rats through the inhibition of hepatic gluconeogenesis and the activation of hypothalamic Akt via membrane receptors MT1 and MT2 [275]. Another study in rats with 10 mg/kg revealed that chronic melatonin administration at pharmacological doses was able to increases Ca^2+^ levels in lots of organs and tissues, such as liver, pancreas, muscle and white adipose tissues, resulting in the improved insulin sensitivity and secretion, indicating the potential clinical use of melatonin against type 2 diabetes [11]. In addition, melatonin showed its protective capability on myocardial cells in type 2 diabetic rats by reducing oxidative stress and ER stress (induced by the elevated blood glucose) through the activation of SIRT1 signaling pathway and inactivation of protein kinase-like endoplasmic reticulum kinase (PERK)/eukaryotic translation initiation factor 2 (eIF2α)/ATF4 (activating transcription factor 4) signaling pathway [276]. Besides, melatonin could prevent pancreatic islet failure caused by β-cell loss and dysfunction in type 2 diabetes, by attenuating β-cell apoptosis, improving its function, and prolonging its survival through the activation of β-cell melatonin signaling [277]. Recently, melatonin could improve the impaired memory caused by diabetes at 10 mg/kg in rats by improving neurogenesis, synaptogenesis in hippocampi, increasing the receptors of melatonin and insulin, and restoring the downstream signaling pathway for insulin [278]. Additionally, it was found that melatonin (250 μg, i.p.) could accelerate bone healing in rats with diabetes due to its antioxidant properties [279]. Furthermore, melatonin was also observed to restore the endothelial dysfunction and improve vascular responses in diabetic rats with 10 mg/kg administration [271]. 

### 3.8. Anti-Obese Activities

Data from WHO shows that more than 39% of adults (1.9 billion) were overweight and over 13% (600 million) were obese in 2014; meanwhile, 41 million children under the age of 5 were overweight or obese [280]. Obesity is also a strong risk factor for other metabolic diseases. Although obesity is a result of imbalance of energy intake and expenditure, many other factors are involved in and contribute to the obese conditions, such as chronic inflammation, oxidative stress, circadian disruption and sleep deprivation [281]. Melatonin has been reported to attenuate the damage caused by obesity. 

Melatonin could play an important role in energy metabolism in obese mice based on gene modulatory effects, antioxidant and anti-inflammatory capacity. An in vivo study demonstrated that melatonin at 10 mg/kg could induce white adipose tissue browning in rats with obesity-related type 2 diabetes by increasing the uncoupling protein 1 (UCP1) and PGC-1α, the thermogenic proteins, which possibly provided the reasons why melatonin was considered as a contributor to control body weight without effects on food intake and physical activity levels [282]. Additionally, melatonin was observed to benefit homeostasis of renal glutathione which was overproduced due to oxidative stress in obese rats [283]. Moreover, melatonin could regulate the cytokines such as IL-8, IL-10, IFN-γ, and inducible protein 10, which were considered as predictors of overweight and obesity [284]. It was also observed that insulin, insulin-like growth factor 1 (IGF1), and IL-17 increased in obesity. In addition, IL-17 alone or combined with insulin could promote CXCL1 and CCL20 expression, which could be suppressed by melatonin at 10 nM through inhibiting Akt activation and increasing GSK3β activity [169]. Since the adipocytes produce adipokines, the increase of TNF-α, resistin, and visfatin was found under obese conditions in mice, which melatonin could suppress to some degree when used at 100 mg/kg, indicating the beneficial impact of melatonin to control obesity [285]. 

It has been reported that that both clock genes [286] and metabolic genes [287] were identified in adipose tissues, which could regulate lipid generation and metabolism, adipocyte proliferation and differentiation, and adipose endocrine functions. Moreover, a cohort study of women aged 16 and above in the UK revealed that light at night (LAN) was significantly associated with obesity, indicating melatonin was involved in the development of obesity due to its regulatory effects on sleep and circadian rhythms [288]. Furthermore, melatonin could promote circadian rhythm-mediated proliferation in adipose tissue in mice at 20 mg/kg by increasing the expression of adipocyte proliferation genes via a complex of Clock/histone deacetylase 3 (HDAC3)/c-Myc [206]. 

Melatonin was also reported to prevent the harmful effects induced by obesity on the main functional organs to reduce the complications of obesity. It was found that melatonin at 4 mg/kg could exhibit the cardioprotective effects in rats with diet-induced obesity as shown by the decreased myocardial infarct sizes and insulin resistant, and the increased serum adiponectin, protein kinase B (PKB)/Akt, ERK42/44, GSK-3β and STAT3 without affecting the body weight or visceral adiposity [289]. Melatonin could also prevent renal failure at the dose of 100 mg/kg through improving the morphology and the functions of renal convoluted tubules in obese mice, by increasing mitofusin-2 expression, an apoptosis modulator [290]. In addition, when used at 10 mg/kg, melatonin was proved to markedly improve non-alcoholic liver steatosis in mice with obesity induced by high fat diet, presenting the decreased TNF-α, IL-1β, IL-6, and phosphorylation of p38 and JNK1/2, indicating the MAPK-JNK/p38 signaling pathway was involved [291]. 

### 3.9. Neuroprotective Activities

Melatonin was found to exhibit the neuroprotective effects on CNS [292]. In a mouse model of traumatic brain injury (TBI), melatonin was administered by 10 mg/kg i.v. at 0, 1, 2, 3, and 4 h post-TBI, the pathological alternations were significantly ameliorated, such as cortical neuronal degeneration and brain edema, mainly due to its antioxidant property through the Nrf2-ARE (antioxidant responsive element) pathway [293]. It was also reported that melatonin could prevent the adult mice brain from IRI at the dose of 10 mg/kg, administrated intraperitoneally, twice (immediately after induction of ischemia and at reperfusion onset). The suggested underling mechanism was to restore the function of mitochondrial by activating SIRT1 signaling pathway [294]. Another research in rats with early brain injury following a hemorrhagic stroke, in particular subarachnoid hemorrhage (SAH), found that melatonin (150 mg/kg, i.p.) could markedly alleviate brain edema, restore the integrity of blood-brain barrier (BBB) by suppressing cortical expressions of proinflammatory cytokines (e.g., IL-1β, IL-6, and TNF-α) [295]. Besides, it was observed in mice that lower dose of melatonin (5 mg/kg, i.p.) could also reduce the injuries in both gray and white matter and better the neurobehavioral outcomes induced by transient focal cerebral ischemia as a potent radical scavenger [296]. Furthermore, melatonin (10 mg/kg) was found to exhibit the neuroprotective and anti-apoptotic effects on oxidative brain damage induced by hemolytic hyperbilirubinemia in newborn Sprague-Dawley rats by reversing the increased plasma TNF-α, IL-1β levels, as well as the decreased brain derived neurotrophic factor (BDNF), S100 calcium-binding protein B (S100B) and IL-10 values [297]. 

The benefits that melatonin could provide to the peripheral nerve have also been widely investigated. It was found that the diclofenac sodium (DS) exposure during pregnancy could damage the fetal peripheral nerve system in rats, which could be improved by melatonin at doses of 10 and 50 mg/kg, showing the significant increase in the axon numbers of right sciatic nerve in male newborn rats with both of the doses administrated. Moreover, the significant increase in the diameter of the axons was found only with high dose of melatonin [298]. Additionally, optic neuritis (ON) might induce permanent vision loss, characterized by inflammation, demyelination, and neurodegeneration of the optic nerve. Melatonin showed its preventive effects by subcutaneous pellet of 20 mg in adult male Wistar rats with ON induced by LPS. The mechanism might be preventing the decrease in visual evoked potentials (VEPs) and pupil light reflex (PLR), inhibiting microglial reactivity, astrocytosis, demyelination, and axon and retinal ganglion cell loss and preserving anterograde transport of cholera toxin β-subunit from the retina to the superior colliculus. In the same study, the therapeutical effects of melatonin on ON was also observed as it reversed the decrease in VEPs and PLR completely when the pellet was implanted at 4 days postinjection of LPS [299]. 

Since reduced melatonin concentrations were identified in chronic neurodegenerative disease, such as Alzheimer’s disease (AD) and Parkinson’s disease (PD), exogenous melatonin treatment showed excellent neuroprotective effects as melatonin is a member of neuroimmuno-endocrine regulator family, which is involved in circadian rhythm, immune and redox actions and also attributing to its potent capacity of anti-apoptosis, and anti-inflammation [300,301]. It was found that the decreased melatonin concentrations in blood were significantly correlated to the loss of hypothalamic gray matter volume and disease severity in PD patients [302]. In addition, it was reported that the melatonin at 4 mM could improve sleep disorders and synaptic dysfunction in Drosophila caused by human leucine-rich repeat kinase 2 (hLRRK2), the most common genetic factor of PD [303]. For AD, amyloid-beta (Aβ) is one of the key pathological factors, which could initiate of the cognitive symptoms of dementia and its aggregation could result in synaptic loss, inflammation and cell death [304]. It was reported that melatonin administration at 10 mg/kg could significantly restore mRNA and protein levels of β-APP-cleaving enzyme 1 (BACE1) and presenilin 1 (PS1) in aged mice [305]. In addition, melatonin (500 mg/kg in 25% ethanol, i.p.) was found to improve the injured spatial learning and memory induced by Aβ1–42, restore the synaptic plasticity, and attenuate astrogliosis in rat. In addition, the in vitro study in primary hippocampal neuron demonstrated that melatonin (50 μM) exhibited the neuroprotective effects against Aβ1–42 through the Musashi1/Notch1/Hairy and enhancer of split 1(Hes1) signaling pathway [301]. 

Besides, it is quite common that nerve injuries can also be induced by medications, toxic chemicals, irradiation or other causes. Oxaliplatin (Oxa) can cause neurotoxicity as a third-era platinum-based chemotherapy against colorectal cancers. The outcomes of 10 mg/kg i.p. melatonin pretreament in rats followed by Oxa injections (4 mg/kg, i.p.) showed that the motor activity and muscular strength were improved, presenting that the inactivation of Bcl-2, caspase 3 apoptotic protein and cyt c release were modulated as well as the non-enzymatic, enzymatic antioxidants and complex enzymes of mitochondria [306]. Additionally, melatonin was reported to prevent neurotoxicity induced by cadmium in mouse neuroblastoma cells, via the activation of transcription factor EB-dependent autophagy-lysosome [136]. Moreover, the beneficial effects of melatonin against irradiation was observed after melatonin was administered at 100 mg/kg in male Sprague-Dawley rats, due to the caspase-3 inhibition [307]. In addition, melatonin (50 mg/kg) was found to prevent the neuronal damage in the hippocampus in pregnant rats caused by 900 MHz electromagnetic fields (EMF), which might be associated with the use of mobile phones [308]. Furthermore, it has been suggested that if the *N*-acetyl group of melatonin was removed, its antioxidant and neuroprotective properties would be improved at the expense of toxic methamphetamine-like effects in several cell lines such as HT22 cells [309].

### 3.10. Other Bioactivities

Melatonin and its receptors are widely distributed in the body, which were identified in numerous organs, tissues, cells and subcellular compartment, such as brain, lung, muscles, bones, renal cells, reproductive cells, mitochondria and nuclei. It has been well documented that melatonin exerts beneficial effects on a large variety of diseases [310]. In terms of bones and muscles, it was suggested that melatonin supplementation of 10 mg/kg could counteract age-related bone loss in rats by improving the microstructure and biomechanical properties of aged bones [311]. Melatonin was also observed to improve muscle function in the animal model of Duchenne muscular dystrophy by causing the dystrophic muscle to contract and relax faster via decreasing plasma creatine kinase activity, providing a better redox status of the muscle [312], and normalizing plasma pro-inflammatory cytokines [150]. In addition, some in vitro study suggested that melatonin could represent a potential therapeutic impact on chronic respiratory diseases, e.g., asthma and chronic obstructive pulmonary diseases, in a dose-dependent manner, by inhibiting mucin 5AC production via the suppression of MAPK signaling in human airway epithelial cells [313]. Besides, a number of studies using rat models showed that melatonin could not only provide protection against acute kidney injury and partially reverse it [314], but also exert its protective effects on chronic kidney disease by scavenging free radicals [315], attenuating the chronic inflammation and limiting apoptosis [316]. Furthermore, an in vitro study found that melatonin pretreatment (100 μM) of human adipose tissue-derived mesenchymal stromal cells could improve their renoprotective and prosurvival effects on human kidney cells [317]. Moreover, it was found that the melatonin levels during pregnancy and labor were elevated and significantly higher than those after birth [318], and studies on pregnant rats indicated that melatonin interacted with other hormones and played a role as a triggering labor contributor [319]. Additionally, melatonin was able to attenuate the inhibitory effects of ROS on fertilization in both men and women [320,321]. It was also demonstrated in vivo that melatonin was closely associated with the health of oral cavity, presenting reducing damage caused by oxidative stress [322], suppressing inflammation, stimulating the proliferation of collagen and osseous tissue, improving wound healing [323] and preventing oral cancer [16]. Furthermore, several researches have shown that melatonin could contribute to a healthy aging and an extended lifespan, and a recent review has summarized the studies regarding the antiaging properties of melatonin [324]. 

In general, bioactivities of melatonin are commonly investigated with high purity in laboratory. For human beings, as the endogenous melatonin alters all through the life, the dietary intake might partially recompense the declined endogenous melatonin after puberty. While for the aged people, supplements might be more suitable, and even medicine could be applied for patients with sleep disorders.

### 3.11. Adverse Effects

No adverse effects has been observed by the consumption of melatonin in foods or drinks. In addition, 1–10 mg/kg is usually considered as standard dose for assisting sleep and there were no toxicological effects found at a dose of 10 mg melatonin (orally) in a 28-day randomized, double-blind clinical trial [326,327]. Furthermore, no toxic effects were observed with high-dose melatonin in pregnant animal models [328]. 

Nevertheless, some adverse effects, such as dizziness, headache, nausea and sleepiness, have been reported by administration of high-dose melatonin, which was used as treatment of some diseases [329,330]. In a case of melatonin overdose (oral administration), lethargy and disorientation occurred to a man of 66 years old caused by 24 mg/kg melatonin for relaxation and sleep before operation, though he recovered completely afterwards [331]. In addition, the results of research on intravenous administration of melatonin appeared not consistent. Some studies found no side effects when high-dose melatonin (i.v.) was used repeatedly to treat pain [332], sepsis [333], surgical procedures [334] and lung disease [335]. Other studies reported that minor signs of sedation, impaired psychomotor and disorientation could possibly be associated with melatonin (i.v.) [336,337]. Although melatonin shows its protective and pro-fertilization effects on reproductive organs in both men and women, puberty seems like a sensitive period for melatonin administration regardless of sex. As the exogenous melatonin was given to male rats, reduced testis size and suppressed sperm production were observed, which were reversed by the treatment with exogenous gonadotropins [338]. For female rats, delayed puberty onset were observed with exogenous melatonin as a result of luteinizing hormone and prolactin reduction [14,339]. Those results were because of the inhibitory effects of melatonin on gonadotropin-releasing hormone neurons in hypothalamus [340]. 

## 4. Conclusions

Melatonin has been identified and qualified in a large number of foods. The content of melatonin is higher in eggs and fish than that in meat in animal foods, while in plant foods, the highest contents of melatonin was found in nuts, and some cereals and germinated legumes or seeds are also rich in melatonin. Mushrooms are also good dietary sources of melatonin. In addition, the intake of melatonin containing foods could significantly increase the melatonin concentration in human serum, indicating melatonin could provide beneficial effects on health through foods. Studies have shown that melatonin has many bioactivities, such as antioxidant, anti-inflammatory, enhancing immunity, anticancer, improving circadian cycle, cardiovascular protecting, anti-diabetic, anti-obese, anti-aging and neuroprotection. Therefore, the consumption of foods rich in melatonin could not only improve insomnia, which affects one third of the general population worldwide, but also provide other health benefits. In the future, the content of melatonin is worth testing and evaluating in more foods to find new natural sources of melatonin, and the mechanisms of action are needed to be investigated more comprehensively. In addition, more clinical trials are necessary to be conducted to clarify the effects of melatonin on human beings. Meanwhile, some foods with extremely high content of melatonin are of great value to be developed into functional foods, which would contribute to the prevention and treatment of various diseases.

## Figures and Tables

**Table 1:** Concentration of melatonin in food.

Name	Scientific Name/Variety/Origin	MT Value or Range ng/g or pg/mL	No. of Samples	Quantified by	Reference
**Animal Foods**					
*Meat*					
Lamb	Not specified	1.6 ± 0.14 ng/g	5	HPLC	[22]
Beef	Not specified	2.1 ± 0.13 ng/g	5	HPLC	[22]
Pork	Not specified	2.5 ± 0.18 ng/g	5	HPLC	[22]
*Fish*					
Salmon	Not specified	3.7 ± 0.21 ng/g	5	HPLC	[22]
*Chicken*					
Meat and skin	Not specified	2.3 ± 0.23 ng/g	5	HPLC	[22]
Liver and heart	Not specified	1.1 ± 0.01 ng/g	5	HPLC	[22]
*Egg*					
Dried solids	Not specified	6.1 ± 0.95 ng/g	5	HPLC	[22]
Raw, whole	Not specified	1.54 ng/g	5	HPLC	[22]
*Milk and dairy products*					
Human milk					
Indian human milk	Not specified	15.92 ± 1.02 pg/mL	6	LC-MS/MS	[37]
Breast milk	Not specified	0–42 pg/mL	5	ELISA	[41]
Bovine milk					
Fresh colostrum,	Not specified	0.06 ng/g	5	HPLC	[22]
Cow milk	Not specified	14.45 ± 0.12 pg/mL	6	LC-MS/MS	[37]
Cow Milk	Holstein cows	4.03–39.43 pg/mL	3	EIA	[38]
Colostrum powder	Not specified	0.6 ± 0.06 ng/g	5	HPLC	[22]
Toned milk	Not specified	18.41 ± 0.62 pg/mL	6	LC-MS/MS	[37]
Yoghurt	Not specified	0.13 ± 0.01 ng/mL	5	LC-MS/MS	[42]
Artificial formulas	Not specified	nd	15	ELISA	[41]
Fermented milk drink	Kefir	nd	5	LC-MS/MS	[42]
**Plant foods**					
*Cereals*					
Corn					
Corn (whole, yellow)	Not specified	1.3 ± 0.28 ng/g	5	HPLC	[22]
Corn (germ meal)	Not specified	1.0 ± 0.10 ng/g	5	HPLC	[22]
Corn (YM001-)	58 cultivars	10–2034 ng/g DW	N/A	HPLC	[31]
Corn	Not specified	1.88 ng/g FW	N/A	GC/MS	[47]
Sweet corn	Not specified	1.37 ng/g FW	N/A	HPLC-FD	[83]
Rice					
Rice	*Oryza sativum* L. ssp. japonica	1.50 ng/g FW	N/A	GC/MS	[47]
Rice (SD001-)	25 cultivars	0–264 ng/g DW	N/A	HPLC	[31]
Black glutinous	Long grain, waxy, Thailand (Bran)	73.81 ± 0.07 ng/g DW	9	PLE HPLC-FD	[17]
Black	Not specified	182.04 ± 1.62 ng/g DW	9	PLE HPLC-FD	[17]
Red	Not specified	212.01 ± 1.37 ng/g DW	9	PLE HPLC-FD	[17]
Whole short grain	Not specified	47.83 ± 0.12 ng/g DW	9	PLE HPLC-FD	[17]
Whole semi-long grain	Not specified	42.95 ± 0.64 ng/g DW	9	PLE HPLC-FD	[17]
Polished short grain	Not specified	31.99 ±0.31 ng/g DW	9	PLE HPLC-FD	[17]
Polished long grain	Not specified	27.61 ± 1.16 ng/g DW	9	PLE HPLC-FD	[17]
Basmati	Not specified	38.46 ± 0.07 ng/g DW	9	PLE HPLC-FD	[17]
Parboiled rice	Not specified	28.33 ± 0.61 ng/g DW	9	PLE HPLC-FD	[17]
Rice	*Oryza sativa* cv. Dongjin	0.04 ng/g DW	20	HPLC	[84]
Rice (transgenic)	*Oryza sativa* cv. Dongjin	0.07–1.25 ng/g DW	20	HPLC	[84]
Rice	*Oryza sativum* L. ssp. japonica	1.01 ng/g FW	N/A	HPLC-FD	[83]
Wheat					
Wheat	*Triticum aestivum* L.	124.7 ± 14.9 ng/g FW	N/A	HPLC-ECD	[85]
Whole grain	Not specified	2–4 ng/g	N/A	Not specified	[86]
Purple wheat	Not specified	4 ng/g DW	3	HPLC-UV	[87]
Purple (heat stressed)	Not specified	2 ng/g DW	3	HPLC-UV	[87]
Barley					
Barley	*Hordeum vulgare* L.	0.87 ng/g FW	N/A	GC/MS	[47]
Barley	*Hordeum vulgare* L.	82.3 ± 6.0 ng/g FW	N/A	HPLC-ECD	[85]
Barley	*Hordeum vulgare* L.	0.38 ng/g FW	N/A	HPLC-FD	[83]
Oats					
Oats	*Avena sativa* L.	90.6 ± 7.7 ng/g FW	N/A	HPLC-ECD	[85]
Oat	*Avena sativa* L.	1.80 ng/g FW	N/A	HPLC-FD	[83]
Bread					
Crumb	Specific ingredients	0.19–0.63 ng/g DW	3	LC-ESI-MS/MS	[20]
Crumb	Not specified	0.34 ± 0.03 ng/g DW	5	LC-MS/MS	[42]
Crust	Specific ingredients	0.14–0.82 ng/g DW	3	LC-ESI-MS/MS	[20]
Crust	Not specified	0.14 ± 0.02 ng/g DW	5	LC-MS/MS	[42]
*Fruits*					
Pineapple					
Pineapple	*Ananas comosus* L.	0.28 ng/g FW	N/A	GC/MS	[47]
Pineapple	*Ananas comosus* L.	0.04 ng/g FW	N/A	HPLC-FD	[83]
Kiwi fruit	*Actinidia chinensis* L.	0.02 ng/g FW	N/A	HPLC-FD	[83]
Strawberry					
Strawberry	*Fragaria magna* L.	0.14 ng/g FW	N/A	GC/MS	[47]
Strawberry	*Fragaria ananassa* L. cv. Camarosa	5.58 ± 0.01 ng/g FW	3	LC-MS/LC-FD	[44]
Strawberry	*Fragaria ananassa* L. cv. Candonga	5.5 ± 0.6 ng/g FW	3	LC-MS/LC-FD	[44]
Strawberry	*Fragaria ananassa* L. cv. Festival	11.26 ± 0.13 ng/g FW	3	LC-MS/LC-FD	[44]
Strawberry	*Fragaria ananassa* L. cv. Primoris	8.5 ± 0.6 ng/g FW	3	LC-MS/LC-FD	[44]
Strawberry	*Fragaria magna* L.	0.01 ng/g FW	N/A	HPLC-FD	[83]
Banana	*Musa ensete*	0.66 ng/g FW	N/A	GC/MS	[47]
Apple					
Apple	*Malus domestica*	nd	N/A	HPLC-FD	[83]
Apple	*Malus domestica Borkh*. cv. Red Fuji	5 ng/g FW	3	HPLC	[88]
Apple	*Malus domestica*	0.16 ng/g FW	N/A	GC/MS	[47]
Pomegranata	*Punica granatum*	0.17 ng/g FW	N/A	GC/MS	[47]
Mulberry					
Mulberry	*Hongguo2 Morusnigra*, black	1.41 ng/g FW	3	HPLC-ESI-MS/MS	[36]
Mulberry	*Baiyuwang Morus alba*, white	0.58 ng/g FW	3	HPLC-ESI-MS/MS	[36]
Cherry					
Tart cherries (frozen)	*Prunus cerasus* L. cv. Balaton	2.9 ± 0.6 ng/g DW	3	HPLC-EMS	[60]
Tart cherries (dry)	*Prunus cerasus* L. cv. Balaton	nd	3	HPLC-EMS	[60]
Tart cherries	*Prunus cerasus* L. cv. Balaton	13.46 ± 1.10 ng/g FW	3	HPLC-ECD	[46]
Tart cherries (frozen)	*Prunus cerasus* L. cv. Montmorency	12.3 ± 2 ng/g DW	3	HPLC-EMS	[60]
Tart cherries (dry)	*Prunus cerasus* L. cv. Montmorency	nd	3	HPLC-EMS	[60]
Tart cherries	*Prunus cerasus* L. cv. Montmorency	2.06 ± 0.17 ng/g FW	3	HPLC-ECD	[46]
Cherry	*Prunus avium* L. cv. Hongdeng	10–20 ng/g FW	3	SPE HPLC	[89]
Cherry	*Prunus avium* L. cv. Rainier	10–20 ng/g FW	3	SPE HPLC	[89]
Cherry	*Prunus avium* L. cv. Burlat	0.22 ng/g FW	3	HPLC-MS	[30]
Cherry	*Prunus avium* L. cv. Navalinda	0.03 ng/g FW	3	HPLC-MS	[30]
Cherry	*Prunus avium* L. cv. Van	0.01 ng/g FW	3	HPLC-MS	[30]
Cherry	*Prunus avium* L. cv. Pico Limón Negro	0.01 ng/g FW	3	HPLC-MS	[30]
Cherry	*Prunus avium* L. cv. Sweetheart	0.06 ng/g FW	3	HPLC-MS	[30]
Cherry	*Prunus avium* L. cv. Pico Negro	0.12 ng/g FW	3	HPLC-MS	[30]
Cherry	*Prunus avium* L. cv. Ambrunés	nd	3	HPLC-MS	[30]
Cherry	*Prunus avium* L. cv. Pico Colorado	0.05 ng/g FW	3	HPLC-MS	[30]
Grape					
Grape	Albana, white	1.2 ng/g	3	HPLC-FD	[43]
Grape (skin)	*Vitis vinifera* L. cv. Merlot	9.3 ± 0.14 ng/g grapes	3	UPLC-MS/MS	[90]
Grape (fresh)	*Vitis vinifera* L. cv. Merlot	3.9 ± 0.06 ng/g grapes	3	UPLC-MS/MS	[90]
Grape (skin)	*Vitis vinifera* L. cv Malbec	8.9–158.9 ng/g DW	3	HPLC-ESI-MS/MS	[45]
Grape (skin)	*Vitis vinifera* L. cv. Nebbiolo	0.97 ng/g	3	HPLC-ELISA	[35]
Grape (skin)	*Vitis vinifera* L. cv. Croatina,	0.87 ng/g	3	HPLC-ELISA	[35]
Grape (skin)	*Vitis vinifera* L. cv. Barbera	0.63 ng/g	3	HPLC-ELISA	[35]
Grape (skin)	*Vitis vinifera* L. cv. Cabernet Sauvignon	0.42 ng/g	3	HPLC-ELISA	[35]
Grape (skin)	*Vitis vinifera* L. cv. Cabernet Franc	0.01 ng/g	3	HPLC-ELISA	[35]
Grape (skin)	*Vitis vinifera* L. cv. Marzemino,	0.03 ng/g	3	HPLC-ELISA	[35]
Grape (skin)	*Vitis vinifera* L. cv. Sangiovese	0.33 ng/g	3	HPLC-ELISA	[35]
Grape (skin)	*Vitis vinifera* L. cv. Merlot,	0.26 ng/g	3	HPLC-ELISA	[35]
Grape (skin)	*Vitis vinifera* L. cv. Malbec,	1.2 ng/g	5	CEC	[56]
Grape (skin)	*Vitis vinifera* L. cv. Cabernet Sauvignon	0.8 ng/g	5	CEC	[56]
Grape (skin)	Chardonnay	0.6 ng/g	5	CEC	[56]
Cranberry					
Cranberry	*Vaccinium oxycoccos* L.	40 ± 10 ug/g DW **	5	UPLC-MS	[91]
Cranberry	*Vaccinium vitis-idaea* L.	25 ± 3 ug/g DW **	5	UPLC-MS	[91]
Cranberry	*Vaccinium macrocarpon* Ait.	96 ± 26 ug/g DW **	5	UPLC-MS	[91]
*Vegetables*					
Onion	*Allium cepa* L.	0.30 ng/g FW	N/A	GC/MS	[47]
Onion	*Allium fistulosum* L., Welsh	0.09 ng/g FW	N/A	HPLC-FD	[83]
Onion	*Allium cepa* L.	0.03 ng/g FW	N/A	HPLC-FD	[83]
Garlic	*Allium sativum* L.	0.59 ng/g FW	N/A	GC/MS	[47]
Cabbage	*Brassica oleraceae* L. var. capitata	0.31 ng/g FW	N/A	GC/MS	[47]
Cauliflower	*Brassica oleraceae* L. var. botrytis	0.82 ng/g FW	N/A	GC/MS	[47]
Turnip	*Brassica rapa* L.	0.50 ng/g FW	N/A	GC/MS	[47]
Cucumber	*Cucumis sativus* L.	0.59 ng/g FW	N/A	GC/MS	[47]
Cucumber	*Cucumis sativus* L.	0.03 ng/g FW	N/A	HPLC-FD	[83]
Cucumber	Not specified	0.01 ng/g	1	GC/MS	[48]
Carrot	*Daucus carota* L.	0.49 ng/g FW	N/A	GC/MS	[47]
Carrot	*Paucus carota* L.	0.06 ng/g FW	N/A	HPLC-FD	[83]
Radish	*Raphnus sativus* L.	0.76 ng/g FW	N/A	GC/MS	[47]
Japanese radish	*Bassica campestris* L.	0.66 ng/g FW	N/A	HPLC-FD	[83]
Potato	*Solanum tuberosum* L.	nd	N/A	GC/MS	[47]
Potato	Not specified	nd	1	GC/MS	[48]
Ginger	*Zingiber officinale* Rosc.	1.42 ng/g FW	N/A	GC/MS	[47]
Black olive	Not specified	0.01 ng/g DW	5	LC-MS/MS	[42]
Beetroot	*Beta vulgaris*	0.002 ng/g	1	GC/MS	[48]
Purslane	*Portulaca oleracea* L.	19 ng/g WW	N/A	GC/MS	[92]
Spinach	Not specified	0.04 ng/g WW	N/A	GC/MS	[92]
Indian spinach	*Basella alba* L.	0.04 ng/g FW	N/A	HPLC-FD	[83]
Asparagus	*Asparagus officinalis* L.	0.01 ng/g FW	N/A	HPLC-FD	[83]
Pepper	*Capsicum annuum* L. cv. Barranca	4.48 ng/g FW/31.01 ng/g DW	4	UHPLC-MS/MS	[49]
Pepper	*Capsicum annuum* L. cv. F26	11.9 ng/g FW/93.4 ng/g DW	4	UHPLC-MS/MS	[49]
Tomato	*Solanum lycopersicum* L. cv. Ciliegia	0.64 ng/g FW/7.47 ng/g DW	4	UHPLC-MS/MS	[49]
Tomato	*Solanum lycopersicum* L. cv. Optima	14.77 ng/g FW/249.98 ng/g DW	4	UHPLC-MS/MS	[49]
Tomato	*Solanum lycopersicum* L. cv. Micro-Tom	1.5–66.6 ng/g FW	N/A	EIA	[93]
Tomato	*Lycopersicon pimpinellifolium*	0.11 ng/g	1	GC/MS	[48]
Tomato	*Lycopersicon esculentum Mill*. cv. Sweet 100	0.51 ng/g	1	GC/MS	[48]
Tomato	*Lycopersicon esculentum Mill*. cv. Rutgers California Supreme	0.17 ng/g	1	GC/MS	[48]
Tomatoes	*Lycopersicon pimpinellifolium*	0.30 ng/g FW	N/A	GC/MS	[47]
Tomatoes	*Lycopersicon esculentum* cv. Bonda	23.87 ± 2.02 ng/g FW	3	LC-MS/LC-FD	[44]
Tomatoes	*Lycopersicon esculentum* cv. Borsalinaa	8.2 ± 0.6 ng/g FW	3	LC-MS/LC-FD	[44]
Tomatoes	*Lycopersicon esculentum* cv. Catalinaa	4.1 ± 0.9 ng/g FW	3	LC-MS/LC-FD	[44]
Tomatoes	*Lycopersicon esculentum* cv. Gordala	17.10 ± 1.21 ng/g FW	3	LC-MS/LC-FD	[44]
Tomatoes	*Lycopersicon esculentum* cv. Lucindaa	4.45 ± 0.05 ng/g FW	3	LC-MS/LC-FD	[44]
Tomatoes	*Lycopersicon esculentum* cv. Marbonea	18.13 ± 2.24 ng/g FW	3	LC-MS/LC-FD	[44]
Tomatoes	*Lycopersicon esculentum* cv. Myriadea	8.0 ± 1.3 ng/g FW	3	LC-MS/LC-FD	[44]
Tomatoes	*Lycopersicon esculentum* cv. Pitenzaa	14.0 ± 2.5 ng/g FW	3	LC-MS/LC-FD	[44]
Tomatoes	*Lycopersicon esculentum* cv. Santonioa	7.73 ± 1.22 ng/g FW	3	LC-MS/LC-FD	[44]
Tomatoes (wild type)	*Solanumlycopersicum* L. cv. Micro-Tom	6.58 ng/g FW	3	HPLC	[94]
Tomatoes (transgenic)	*Solanumlycopersicum* L. cv. Micro-Tom	7.39–10.34 ng/g FW	3	HPLC	[94]
Tomatoes	Not specified	0.03 ± 0.01 ng/g DW	5	LC-MS/MS	[42]
Tomato	*Lycopersicon esculentum* L.	0.03 ng/g FW	N/A	HPLC-FD	[83]
Japanese butterbur	*Patasites japonicus 50*	nd	N/A	HPLC-FD	[83]
Taro	*Colocasis escutenta*	0.06 ng/g FW	N/A	HPLC-FD	[83]
Cabbage	*Brassica oleracea*	0.11 ng/g FW	N/A	HPLC-FD	[83]
Chinese cabbage	*Raphamus sativas*	0.11 ng/g FW	N/A	HPLC-FD	[83]
Chungitsu	*Chrysanthemum cororarum*	0.42 ng/g FW	N/A	HPLC-FD	[83]
Ginger	*Zinigiber officinale*	0.58 ng/g FW	N/A	HPLC-FD	[83]
Japanese ashitaba	*Angelica* keiskei	0.62 ng/g FW	N/A	HPLC-FD	[83]
Mushrooms					
Mushroom	Agaricus bisporus*	4300–6400 ng/g DW	3	RP-HPLC	[50]
Basidiomycota	*Armillaria mellea*	<10 ng/g DW	3	HPLC	[19]
Basidiomycota	*Boletus badius*	<10 ng/g DW	3	HPLC	[19]
Basidiomycota	*Boletus edulis*	6800 ± 60 ng/g DW	3	HPLC	[19]
Basidiomycota	*Cantharellus cibarius*	1400 ± 110 ng/g DW	3	HPLC	[19]
Basidiomycota	*Lactarius deliciosus*	12,900 ± 770 ng/g DW	3	HPLC	[19]
Basidiomycota	*Pleurotus ostreatus*	<10 ng/g DW	3	HPLC	[19]
*Legumes and seeds (raw)*					
Legumes					
Lentils	*Lens culinaris* L.	0.5 ng/g DW	3	HPLC-MS/MS	[53]
Kidney beans	*Phaseolus vulgaris* L	1.0 ng/g DW	3	HPLC-MS/MS	[53]
Soybean	*Glycine max*	0.45 ± 0.03 ng/g DW	N/A	RIA	[52]
Seeds					
Lupin (seed-cotyledons)	*Lupinus albus* L.	3.83 ± 0.21 ng/g FW	5	HPLC-FD	[32]
Lupin (seed-coat)	*Lupinus albus* L.	37.50 ± 2.3 ng/g FW	5	HPLC-FD	[32]
Lupin (seed-flour)	*Lupinus albus* L.	0.53 ± 0.04 ng/g DW	5	HPLC-FD	[32]
Grape (seed)	*Vitis vinifera* L. cv. Merlot	10.04 ± 0.49 ng/g grapes	3	UPLC-MS/MS	[90]
Barley (seed)	*Hordeum vulgare* L.	0.58 ± 0.05 ng/g FW	5	HPLC-FD	[32]
Barley(seed-flour)	*Hordeum vulgare* L.	0.09 ± 0.01 ng/g DW	5	HPLC-FD	[32]
Black mustard	*Brassica nigra*	129 ng/g DW	2	HPLC-ECD	[51]
White mustard	*Brassica hirta*	189 ng/g DW	2	HPLC-ECD	[51]
Fenugreek	*Trigonella faena-graecum*	43 ng/g DW	2	HPLC-ECD	[51]
Milk thistle	*Silybum marianum*	2 ng/g DW	2	HPLC-ECD	[51]
Celery	*Apium gravolens*	7 ng/g DW	2	HPLC-ECD	[51]
Alfalfa	*Medicago sativa*	16 ng/g DW	2	HPLC-ECD	[51]
Coriander	*Coriandrum sativum*	7 ng/g DW	2	HPLC-ECD	[51]
Green cardamom	*Elettaria cardamomum*	15 ng/g DW	2	HPLC-ECD	[51]
Fennel	*Foeniculum vulgare*	28 ng/g DW	2	HPLC-ECD	[51]
Poppy	*Popaver somniferum*	6 ng/g DW	2	HPLC-ECD	[51]
Anise	*Pimpinella anisum*	7 ng/g DW	2	HPLC-ECD	[51]
Sunflower	*Helianthus annuus*	29 ng/g DW	2	HPLC-ECD	[51]
Flax	*Linum usitatissimum*	12 ng/g DW	2	HPLC-ECD	[51]
Almond	*Prunus amydalus*	39 ng/g DW	2	HPLC-ECD	[51]
Chinese wolfberry	*Lycium barbarum*	103 ng/g DW	2	HPLC-ECD	[51]
Cucumber	*Cucumissativus* L. cv. Jingyu-1	5.1 ng/g FW	3	UPLC-ESI-MS/MS	[95]
Alfalfa	*Medicago sativa* L.	0.05 ± 0.00 ng/g DW	3	ELISA	[23]
Lentil	*Lens sculenta* L.	0.07 ± 0.01 ng/g DW	3	ELISA	[23]
Mung bean	*Vignaradiata* L.	0.01 ± 0.0 ng/g DW	3	ELISA	[23]
Onion	*Allium cepa* L.	0.22 ± 0.01 ng/g DW	3	ELISA	[23]
Broccoli	*Brassica oleraceae* L.	0.41 ± 0.04 ng/g DW	3	ELISA	[23]
Red cabbage	*Brassica oleraceae capitate rubra* L.	0.34 ± 0.04 ng/g DW	3	ELISA	[23]
Radish (mixed)	*Raphanus sativus japonicum* L.	0.28 ± 0.01 ng/g DW	3	ELISA	[23]
	*Raphanus sativus rambo* L.				
	*Raphanus sativus sinicum rosae* L.				
*Legumes and seeds (germination)*					
Legumes sprouts					
Lentils	*Lens culinaris* L.	1089.8 ng/g DW	3	HPLC-MS/MS	[53]
Lentil	Not specified	0.92 ± 0.06 ng/g DW	N/A	RIA	[52]
Kidney beans	*Phaseolus vulgaris* L.	529.1 ng/g DW	3	HPLC-MS/MS	[53]
Soya bean	*Glycine max* L.	1.89 ± 0.11 ng/g DW	N/A	RIA	[52]
Vetch	*Vicia sativa* L.	1.91 ± 0.11 ng/g DW	N/A	RIA	[52]
Seedling					
Rice	*Oryza sativa* cv. Dongjin	1.9 ng/g DW	3	HPLC	[96]
Rice (transgenic)	*Oryza sativa* cv. Dongjin	2.7–5.2 ng/g DW	3	HPLC	[96]
Cucumber	*Cucumis sativus* L. cv. Jingyu-1	17.3 ng/g FW	3	UHPLC-ESI-MS/MS	[95]
Alfalfa	*Medicago sativa* L.	0.13 ± 0.01 ng/g DW	3	ELISA	[23]
Lentil	*Lens sculenta* L.	0.22 ± 0.0 1 ng/g DW	3	ELISA	[23]
Mung bean	*Vignaradiata* L.	0.17 ± 0.01 ng/g DW	3	ELISA	[23]
Onion	*Allium cepa* L.	0.30 ± 0.02 ng/g DW	3	ELISA	[23]
Broccoli	*Brassica oleraceae* L.	0.44 ± 0.01 ng/g DW	3	ELISA	[23]
Red cabbage	*Brassica oleraceae capitate* rubra L.	0.86 ± 0.05 ng/g DW	3	ELISA	[23]
Radish	*Raphanus sativus japonicum* L.	0.54 ± 0.04 ng/g DW	3	ELISA	[23]
	*Raphanus sativus rambo* L.				
	*Raphanus sativus sinicum rosae* L.				
*Nuts*					
Pistachio					
Pistachio	*Pistacia vera* L. cv. Ahmad Aghaei	233,000 ng/g DW	N/A	GC/MS	[28]
Pistachio	*Pistacia vera* L. cv. Akbari, Kalle	226,900 ng/g DW	N/A	GC/MS	[28]
Pistachio	*Pistacia vera* L. cv. Qouchi	231,400 ng/g DW	N/A	GC/MS	[28]
Pistachio	*Pistacia vera* L. cv. Fandoghi	228,400 ng/g DW	N/A	GC/MS	[28]
Walnuts					
Walnuts	*Juglans regia* L. cv. Serr	1.02 ± 0.06 ng/g FW	4	HPLC–MS	[97]
Walnuts	*Juglans regia* L. cv. Hartley	1.77 ± 0.14 ng/g FW	4	HPLC–MS	[97]
Walnuts	*Juglans regia* L. cv. Chandler	1.37 ± 0.37 ng/g FW	4	HPLC–MS	[97]
Walnuts	*Juglans regia* L. cv. Howard	1.9 ± 0.4 ng/g FW	4	HPLC–MS	[97]
Walnuts	Not specified	0.14 ± 0.03 ng/g DW	5	LC–MS/MS	[42]
Walnuts	*Juglans regia* L.	3.5 ± 1.0 ng/g	5	HPLC-ECD	[78]
*Juices and beverages*					
Beer	Not specified	0.09 ± 0.01 ng/mL	5	LC-MS/MS	[42]
Wine					
Albana must	Albana, Romagna	1.1 ng/mL	3	HPLC-FD	[43]
Albana wine	Albana, Romagna	0.6 ng/mL	3	HPLC-FD	[43]
Albana grappa	Albana, Romagna	0.3 ng/mL	3	HPLC-FD	[43]
Red wine	Not specified	0.26 ± 0.18 ng/mL	3	UHPLCMS/MS	[59]
Dessert ice wine	Not specified	0.17 ± 0.11 ng/mL	3	UHPLCMS/MS	[59]
Groppello wines	*Vitis vinifera* L. cv. Groppello Gentile	5.2 ng/mL	3	UHPLCMS/MS	[59]
Merlot wines	Merlot	8.1 ng/mL	3	UHPLCMS/MS	[59]
Wine	Cabernet Sauvignon	14.2 ± 0.2 ng/mL	3	HPLC-MS/MS	[55]
Wine	Cabernet Sauvignon	0.23 ± 0.01 ng/mL	3	ELISA	[55]
Wine	Jaen Tinto	nd	3	HPLC-MS/MS	[55]
Wine	Jaen Tinto	0.16 ± 0.01 ng/mL	3	ELISA	[55]
Wine	*Vitis vinifera* L. cv. Merlot	nd	3	HPLC-MS/MS	[55]
Wine	*Vitis vinifera* L. cv. Merlot	0.21 ± 0.02 ng/mL	3	ELISA	[55]
Wine	Palomino Negro	nd	3	HPLC-MS/MS	[55]
Wine	Palomino Negro	0.28 ± 0.00 ng/mL	3	ELISA	[55]
Wine	Petit Verdot	5.1 ± 0.6 ng/mL	3	HPLC-MS/MS	[55]
Wine	Petit Verdot	0.22 ± 0.01 ng/mL	3	ELISA	[55]
Wine	Prieto Picudo	49.0 ± 4.7 ng/mL	3	HPLC-MS/MS	[55]
Wine	Prieto Picudo	0.19 ± 0.01 ng/mL	3	ELISA	[55]
Wine	Syrah	86.5 ± 2.6 ng/mL	3	HPLC-MS/MS	[55]
Wine	Syrah	0.22 ± 0.02 ng/mL	3	ELISA	[55]
Wine	Tempranillo	129.5 ± 3.5 ng/mL	3	HPLC-MS/MS	[55]
Wine	Tempranillo	0.14 ± 0.01 ng/mL	3	ELISA	[55]
Wine	*Vitis vinifera* L. cv. Malbec	0.24 ng/mL	5	CEC	[56]
Wine	Cabernet Sauvignon	0.32 ng/mL	5	CEC	[56]
Wine	Chardonnay	0.16 ng/mL	5	CEC	[56]
Wine	Sangiovese, red	0.5 ng/mL	3	HPLC-FD	[43]
Wine	Trebbiano, white	0.4 ng/mL	3	HPLC-FD	[43]
Coffee beans					
Green coffee	Not specified	0.040 ± 0.01 ng/g DW	5	LC-MS/MS	[42]
Green beans	*Coffea canephora* L. (robusta)	5800 ± 800 ng/g DW	3	LC-MS-ESI	[58]
Green beans	*Coffea arabica* L. (arabica)	6800 ± 400 ng/g DW	3	LC-MS-ESI	[58]
Roasted beans	*Coffea canephora* L. (robusta)	8000 ±.900 ng/g DW	3	LC-MS-ESI	[58]
Roasted beans	*Coffea arabica* L. (arabica)	9600 ± 800 ng/g DW	3	LC-MS-ESI	[58]
Decoction (Brew)	*Coffea canephora* L. (robusta)	60 ± 12 ng/mL	3	LC-MS-ESI	[58]
Decoction (Brew)	*Coffea arabica* L. (arabica)	78 ± 5 ng/mL	3	LC-MS-ESI	[58]
Juices					
Orange juice	*Citrus sinensis* L. var. Navel late (Huelva, Spain)	3.15–21.80 ng/mL	3	UHPLC-QqQ-MS/MS	[98]
Grape juice	Not specified	0.5 ng/mL	3	HPLC-FD	[43]
Cacao powder	Not specified	0.01 ng/g DW	5	LC-MS/MS	[42]
Concentrate					
Tart cherries	*Prunus cerasus* L. cv. Balaton	nd	3	HPLC-EMS	[46]
Tart cherries	*Prunus cerasus* L. cv. Montmorency	nd	3	HPLC-EMS	[46]
Sour cherries	Not specified	nd	5	LC-MS/MS	[42]
Tea					
Green tea	Not specified	nd	5	LC-MS/MS	[42]
Black tea	Not specified	nd	5	LC-MS/MS	[42]
Balsamic vinegars		0.12 ± 0.014 ng/mL	3	UHPLCMS/MS	[59]
*Medical Herbs*					
Huang-qin	*Scutellaria biacalensis*	7110 ng/g DW	2	Not specified	[29]
St John’s Wort (flowers)	*Hypericum perforatum*	4490 ng/g DW	2	Not specified	[29]
Chantui	*Periostracum cicadae*	3771 ng/g DW	3	SPE-HPLC-FD	[61]
Gouteng	*Uncaria rhynchophylla*	2460 ng/g DW	3	SPE-HPLC-FD	[61]
Diding	*Viola philipica Cav*.	2368 ng/g DW	3	SPE-HPLC-FD	[61]
Shiya tea-leaf	*Babreum coscluea*	2120 ng/g DW	3	SPE-HPLC-FD	[61]
Feverfew (fresh leaves)	*Tanacetum parthenium*	1920–2450 ng/g DW	2	Not specified	[29]
St John’s Wort (leaves)	*Hypericum perforatum*	1750 ng/g DW	2	Not specified	[29]
Sangye	*Morus alba* L. (Leaf)	1510 ng/g DW	3	SPE-HPLC-FD	[61]
Huangbo	*Phellodendron amurense* Rupr.	1235 ng/g DW	3	SPE-HPLC-FD	[61]
Sangbaipi	*Mori Albae* (Cortex)	1110 ng/g DW	3	SPE-HPLC-FD	[61]
Yinyanghuo	*Epimedium brevicornum Maxim*	1105 ng/g DW	3	SPE-HPLC-FD	[61]
Black pepper	*Piper nigrum* L.	1092.7 ng/g DW	5	SPE HPLC ELISA	[62]
Huanglian	*Coptis chinensis Franch*	1008 ng/g DW	3	SPE-HPLC-FD	[61]
Mulberry leaves	*Morus* spp. cv. Buriram 60	279.6 ng/g DW	3	HPLC-FD	[99]
	*Morus* spp. cv. Sakonnakhon	100.5 ng/g DW	3	HPLC-FD	[99]
	*Morus* spp. cv. Khunphai	40.7 ng/g DW	3	HPLC-FD	[99]
*Edible oil*					
Virgin Argan oil	Not specified	0.06 ± 0.05 ng/g	2	HPLC-FD	[64]
Refined sunflower	Not specified	0.03–0.08 ng/g	2	HPLC-FD	[64]
Primrose,	Not specified	0.03–0.08 ng/g	2	HPLC-FD	[64]
Refined grape seed	Not specified	0.03–0.08 ng/g	2	HPLC-FD	[64]
Refined walnut	Not specified	0.03–0.08 ng/g	2	HPLC-FD	[64]
Virgin walnut	Not specified	0.03–0.08 ng/g	2	HPLC-FD	[64]
Virgin linseed	Not specified	0.03–0.08 ng/g	2	HPLC-FD	[64]
Linseed oils	Not specified	0.03–0.08 ng/g	2	HPLC-FD	[64]
Refined linseed	Not specified	0.29 ± 0.00 ng/g	2	HPLC-FD	[64]
Virgin sesame	Not specified	0.03–0.08 ng/g	2	HPLC-FD	[64]
Wheat germ	Not specified	0.03–0.08 ng/g	2	HPLC-FD	[64]
Virgin soybean	Not specified	0.19 ± 0.00 ng/g	2	HPLC-FD	[64]
Olive oil	Extra virgin	0.03 ± 0.00 ng/g	2	HPLC-FD	[64]
D.O. Sierra Ma′gina	Not specified	0.11 ± 0.04 ng/mL	3	ELISA	[63]
D.O. Siurana	Not specified	0.10 ± 0.02 ng/mL	3	ELISA	[63]
D.O. Bajo Arago′n	Not specified	0.07 ± 0.02 ng/mL	3	ELISA	[63]
D.O. Montes de Toledo	Not specified	0.11 ± 0.01 ng/mL	3	ELISA	[63]
D.O. Baena	Not specified	0.12 ± 0.00 ng/mL	3	ELISA	[63]
D.O. Sierra de Segura	Not specified	0.09 ± 0.00 ng/mL	3	ELISA	[63]
D.O. Les Garrigues	Not specified	0.10 ± 0.00 ng/mL	3	ELISA	[63]
D.O. Toscano	Not specified	0.11 ± 0.02 ng/mL	3	ELISA	[63]
Refined olive oil sample 1	Not specified	0.05 ± 0.01 ng/mL	3	ELISA	[63]
Refined olive oil sample 2	Not specified	0.08 ± 0.01 ng/mL	3	ELISA	[63]
Refined sunflower oil sample	Not specified	0.05 ± 0.01 ng/mL	3	ELISA	[63]
**Microorganisms**					
Yeast (dried brewer)	*Saccharomyces cerevisae*	2.2 ± 0.14 ng/g	5	HPLC	[22]

Note: All the values are presented in a pattern of mean ± SD. * in vitro cultures grown on media enriched with zinc salts. ** estimated from the figure. Abbreviation used in the table: CEC: capillary electrochromatography; D.O.: designations of origin; ECD: electron capture detector; EIA: enzyme-linked immunosorbent assay; ELISA: enzyme-linked immunoasorbent assay; EMS: electrospray mass spectrometry; ESI: electronic spray ion; FD: fluorescence detector; GC/MS: gas chromatograph/mass spectrum; HPLC: high-performance liquid chromatography; nd: not detected; PLE: pressurized liquid extraction; RIA: radioimmunoassay; RP-HPLC: reversed-phase high-performance liquid chromatography; SPE: solid phase extraction; UHPLC: ultra-high-performance liquid chromatography; UV: ultraviolet.

**Table 2:** Bioactivities and potential mechanisms of melatonin.

Study Type	Subjects	Dose (mg/kg b.w.)/(Dose Dependent)	Potential Mechanisms (Melatonin (Mel) and/or Its Metabolites)	Reference
**Antioxidant Activities**				
			Directly scavenging free radical	
In vivo	Mouse	10 mg/kg b.w.	*Increasing the efficiency of electron transport chain:* - lowering electron leakage and reducing free radical generation	[122]
In vitro	Human umbilical artery segment	10^−6^, 10^−5^, 10^−4^ M (dose dependent)	Significantly scavenging the hydroxyl radical	[127]
			*Cascade effects: removing free radicals efficiently than other reductants:*	
In vivo	Rat	43 μmol/kg b.w.	- more efficient than Vitamin C	[128]
	Rat	2 μmol/kg b.w.	- more efficient than Vitamin E	[125]
	Mouse	5 mg/kg b.w.	- more efficient than Vitamin E	[325]
In vitro	Incubation medium	1–1000 mM	- more efficient than Vitamin C & Vitamin E	[123]
			*Some metabolites more potent than its precursor in reducing oxidative stress:*	
	Fenton reaction-based assay	AFMK: 0.017–0.067 mM AMK: up to 0.2 mM	- the order of efficacy of scavenging ∙OH: AMK > AK > AFMK	[130]
N/A	N/A	N/A	- C3-OHM is 2–3 fold more potent than Mel in reducing hypervalent hemoglobin (Tan & Reiter, unpublished observations).	[131]
			Modulating and activating other enzymes	
			*Downregulating pro-oxidative enzymes*	
In vitro & in vivo	Rat striatum	M & AMK: 10^−11^–10^−3^ M (dose dependent in vitro)	- Mel & AMK inhibiting nNOS activity - AMK more potent in inhibiting nNOS activity than Mel (in vivo)	[142]
In vitro	MCF-7 cells	1 nM	Inhibiting the mRNA expression of COX 1 and COX-2 in MCF-7 cells	[143]
			*Stimulating the synthesis of other antioxidants*	
In vitro	ECV304 cells	1 μM	- Inducing γ-GCS expression to promote GSH synthesis	[124]
In vitro	2 neuronal cell lines: PC12 cells & SK-N-SH	1 nM	- Regulating AOEs gene expression - Increasing mRNA of SODs and GPx	[144]
			*Preventing antioxidant enzymes from oxidative stress*	
In vitro	human BM-MSCs	0 to 1000 μM (dose dependent 10–100 μM)	- significantly restoring SOD (*p* < 0.05) and CAT (*p* < 0.01) - increasing GSH (*p* < 0.01) with pre-treatment of Mel	[145]
In vivo	Sprague-Dawley rats	10 mg/kg b.w.	GSH-Rd activity was completely or partially restored by Mel treatment	[146]
In vitro & in vivo	Sprague–Dawley rats	0–0.1 mM (dose dependent in vitro) 10 mg/kg b.w.	G6PG activity dose-dependent in vitro (increased below 0.08 mM Mel concentration and reached a plateau above 0.1 mM) G6PG activity time-dependent (in vivo)	[147]
			Synergistically working with other reductants	
			Combining with other antioxidants to remove radicals synergistically	
In vitro	Rat liver homogenates	2.5–1600 μM	- dramatically enhancing the protective effects after combining	[148]
**Anti-inflammatory Activities**				
			NF-κB signaling pathway involved mechanisms	
			*Modulating NF-κB and its downstream pro-inflammatory target genes*	
In vitro	Human colon cancer cell lines SW620 and LOVO	1 mmol/L	- iNOS	[70]
In vitro	RAW 264.7 macrophages	0.5, 1, 2 mM (dose dependent)	- COX-2, PGE_2_	[158]
In vitro	Human neuroblastoma dopamine SH-SY5Y cell lines	1, 10, 100 or 1000 nM	- TNF-α	[159]
In vitro	Rat astrocytoma C6 cells	50–200 μM (dose dependent)	- GFAP	[160]
In vitro & in vivo	CHON-001 human chondrocyte cell line Rabbit with osteoarthritis (OA)	0.1, 1, 10, 100 ng (dose- and time-dependent) 20 mg/kg	Protecting cells by blocking the activated NF-κB as well as the phosphorylation of PI3K/Akt, p38, ERK, JNK and MAPK	[161]
In vitro	BV2 murine microglial cell line	1 mM	Downregulating chemokine expression	[162]
In vivo	Rats	5 mg/kg	Inhibiting the inflammatory reaction	[163]
In vivo & in vitro	Female BALB/c mice MMECs	5, 10, 20 mg/kg 25, 50, 100 μM (dose dependent)	Suppressing NF-κB activation and activating PPAR-γ	[164]
In vitro	Mast cells (RBL-2H3)	100 nM and 1 mM (dose dependent)	Inhibiting IKK/NF-κB signal transduction	[165]
			SIRT1 pathway involved mechanisms	
In vitro & in vivo	BV2 cell lysates PND7 rat brain	100 µM 10 mg/kg	Activating SIRT1/Nrf2 signaling pathway to reduce oxidative stress damage	[168]
			Other possible mechanisms	
In vivo	Pediatric patients	10 mg (09:00 h) 60 mg ( 21:00 h )	Regulating the expression of other pro-inflammatory genes	[150]
In vitro	Mouse Gsk3b knockout (Gsk3b^−/−^) and wild-type (Gsk3b^+/+^) MEF cells	10 nM	Inhibiting the expression of inflammatory chemokines/cytokines	[169]
In vivo	Plasmodium	10 µM (time dependent)	Inducing temporal up-regulation of gene expression related to UPS	[170]
In vivo	C57BL mice	10 mg/kg i.p.	Downregulating mRNA of E2F2 and H2-Ab1	[171]
In vivo	Rats	5, 15, and 25 mg/kg (dose-dependent)	Activating the expression of NDRG2, which was involved in cellular differentiation, development, anti-apoptosis, anti-inflammatory cytokine, and antioxidant	[172]
In vivo	Carp	10^−4^–10^−12^ M	Maintaining the pro- and anti- inflammatory balance during infection by influencing leukocyte migration and apoptosis	[151]
**Enhancing Immune Activities**				
			Reciprocally regulating the nervous, endocrine, and immune systems	
In vivo & In vitro	Mice Thymus and spleen cells	4–5 mL/day/mouse 1.5 pg/ml to 1.5 pgg/ml	Regulating thymocyte apoptosis	[174]
In vivo	Mice	1.5 pg/mL to 1.5 pg/mL	The concentration of melatonin correspond with the change of seasons	[175]
			Inhibiting the production of cAMP, cGMP and DAG, and improving the immunity	
In vitro	Human blood lymphocyte	N/A (dose-dependent)	Inhibiting adenylyl cyclase and the stimulating phospholipase C	[183]
In vivo	Golden hamsters	25 μg/100 g/hamster/day	Improving immune responses	[184]
			Protecting the immune organs, tissues and cells	
			*Reversing the weight loss of thymuses and spleens in pinealectomized animals*	
In vivo & In vitro	Mice Thymus and spleen cells	4–5 mL/day/mouse 1.5 pg/mL to 1.5 pg/mL	- thymus	[175]
In vivo	Syrian hamsters	25 μg	- spleen	[188]
In vivo	Pediatric patients	N/A	Increasing tonsillar size	[189]
			*Improving proliferation, increasing activity and inhibiting apoptosis of immune cells*	
In vitro	cultured monocytes	N/A	- monocyte	[190]
In vivo	ICR mice	10 or 50 mg/kg	- natural killer (NK) cells	[191]
In vitro	Neutrophils & peripheral blood mononuclear cells	10 mM	- neutrophils	[192]
In vivo	Wistar albino rats	10 mg/kg	Increasing the sensitivity of the immune cells to some cytokines	[193]
In vitro & In vitro	Thymocytes of Barbari goats Thymus	500 pg/mL 500 pg/mL	Restoring the suppressed immunity of T-cell cultured by developing some hormonal microcircuit (gonadal steroid and melatonin) in lymphatic organs	[194]
			Modulating immune mediator production	
In vitro	Human mononuclear cells	10^−8^ M	Increasing IL-2, IFN-γ and IL-6 in monocytes	[195]
In vitro	Neutrophils & peripheral blood mononuclear cells	10 mM	Mel & AFMK: decreasing IL-8 and TNF-α in neutrophils	[192]
In vitro	RAW264.7 cells	10, 100 or 1000 μM	Decreasing IL-1β, IL-6, IL-8, IL-10 and TNF-α in macrophages	[196]
			Regulating the ROS production in the essential immune cells	
In vitro	Human monocytes	10^−12^ M and above	Activating monocytes (above the activation threshold of 5 × 10^−11^ M)	[199]
In vitro	Lung neutrophils	0.01, 0.1, 1 mM (dose-dependent)	Activating neutrophils	[200]
In vivo	Hamsters	25 μg/100 g b.w.	Attenuating oxidative load	[201]
In vivo	Wild birds	25 μg/100 g/day	Alleviating oxidative damage and suppressing the immune status induced by stress	[202].
In vitro & in vivo	Heart tissue of C57BL/6 C57BL/6J mice	3 or 4 doses of melatonin 30 mg/kg	Suppressing systemic innate immune activation by blocking the NF-κB/NLRP3 connection through a sirtuin1-dependent pathway	[154].
**Improving Circadian Rhythm and Sleep**				
In vivo	C3H & C57BL mice	N/A	Being involved in the control of clock gene protein levels in the adrenal cortex of mice	[211]
In vivo	Soay sheep	N/A	Resetting circadian rhythms in the pituitary pars tuberalis	[212]
In vivo	Mice (C3H/HeJCrl and C57BL/6NCrl)	N/A	Influencing PER1 and CRY2 protein levels Playing a role in rhythmic regulation of pCREB levels in the mammalian retina	[213]
In vitro & in vivo	COS7 cells Lambs	N/A	Activating Npas4	[215]
In vivo	Hamster	20 μg/day	Coordinating the diurnal rhythm in neuronal remodeling	[217]
In vivo	Mice	6 μg/day for 2 weeks	Increasing amplitude in expressional rhythms Altering the expression of genes of serotonergic neurotransmission Improve the depression-like behavior	[218]
In vivo	23 patients	N/A	Being positive correlated with sleep parameters	[220]
**Anticancer Activities**				
			Effects on tumor cell cycle, incl. growth, proliferation, metabolism and apoptosis	
In vitro & In vivo	Human gastric cancer cell lines (AGS and MKN) Male BALB/c nude mice	5 mg/kg/twice/week for 33 days 1 µM to 2 mM (dose-/time- dependent, 15 min to 24 h)	Inhibiting gastric tumor growth and peritoneal metastasis Inhibiting C/EBPβ and NF-κB Inducing ER stress and inhibiting EMT	[232]
In vitro	T47D-BAF co-cultured	20 nM	Suppressing breast cancer cell proliferation and inhibiting aromatase	[238]
In vitro & in vivo	Prostate cancer cells TRAMP male mice	1 mM 200 µg/mL	Reducing glucose uptake and modifying the expression of GLUT1 transporter Attenuating glucose-induced tumor progression and prolonging the lifespan	[233]
In vitro	Hypoxic prostate cancer cell line PC-3 cells	1 mM	Anti-angiogenic property Upregulating miRNA3195 and miRNA 374b and downregulating 16 miRNAs	[239]
In vitro	Colorectal cancer LoVo cells	0.1–2.0 mM (dose-dependent)	Suppressing cell proliferation and inducing apoptosis Inducing dephosphorylation and nuclear import of histone deacetylase 4 (HDAC4) Decreasing H3 acetylation by inactivating CaMKIIα and reducing bcl-2 expression	[240]
In vitro	Breast cancer cell line SK-BR-3 & MDA-MB-231	2 mM	Changing the protein levels of Survivin, Bcl-2, and Bax Affecting cyt c release from the mitochondria to the cytosol Enhancing apoptotic cell death via sustained upregulation of Redd1 expression and inhibition of mTORC1 upstream of the activation of the p38/JNK pathways	[234]
			Effects on invasion and metastasis of tumor cells	
In vitro	HepG2 liver cancer cells	1 mM	Exhibiting anti-invasive and antimetastatic activities by suppressing the activity of MMP-9 Reducing IL-1β-induced HepG2 cells MMP-9 gelatinase activity and inhibiting cell invasion and motility through downregulation of MMP-9 gene expression and upregulation of the MMP-9-specific inhibitor tissue inhibitor of TIMP-1 Suppressing IL-1β-induced NF-κB translocation and transcriptional activity	[241]
In vitro	Renal cell carcinoma cells (Caki-1 and Achn)	0.5–2 mM	Reducing the migration and invasion Inhibiting MMP-9 by reducing p65- and p52-DNA-binding activities Regulating MMP-9 transactivation and cell motility refer to the Akt-mediated JNK1/2 and ERK1/2 signaling pathways	[242]
In vivo & in vitro	Female athymic nude mice Metastatic and non-metastatic breast cancer cell lines (MDA-MB-231)	100 mg/kg/day 1 mM	Lowering the numbers of lung metastasis Decreasing ROCK-1 protein expression in metastatic foci Reducing cell viability and invasion/migration Decreasing ROCK-1 gene expression in metastatic cells and protein expression in non-metastatic cell line	[235]
			Therapy adjunct in tumor treatment	
In vitro	Human non-small-cell lung cancer (NSCLC) cells lines H1299 and A549	1 mM	Enhancing the berberine-mediated growth inhibition of lung cancer cells through simultaneous modulation of caspase/cyt C, AP-2β/hTERT, NF-κB/COX-2, and Akt/ERK signaling pathways	[157]
In vitro	Breast cancer cells	1 nM	Mediating the sensitization to the ionizing radiation by decreasing around 50% the activity and expression of proteins involved in the synthesis of estrogens Reducing the amount of active estrogens at cancer cell level Inducing a 2-fold change in p53 expression compared to radiation alone	[243]
In vivo	Female patients	Melatonin-containing cream for twice daily use	Significantly lowering the occurrence of grade 1/2 acute radiation dermatitis in patients with breast-conserving surgery for stage 0–2 breast cancer	[244]
In vivo	Male Wistar rats	10 mg/kg/week	Mitigating PVB-induced testicular dysfunction	[245]
In vivo & in vitro	Female athymic nude mice Human colon cancer cell lines SW620	25 mg/kg 1 mmol/L	Exerting synergistic anti-tumor effect by inhibiting the AKT and iNOS pathway Enhancing the 5-FU-mediated inhibition of cell proliferation, colony formation, cell migration and invasion Synergizing with 5-FU to promote the activation of the caspase/PARP-dependent apoptosis pathway and induce cell cycle arrest Synergizing anti-tumor effect of 5-FU by targeting the PI3K/AKT and NF-κB/iNOS signaling	[70]
In vitro	Human colorectal cancer cells	N/A	MT2 mRNA expression levels increased The profile of melatonin receptors gene expression and genes associated with their activity in colorectal cancer	[236]
In vitro	Estrogen receptor-positive endometrial cancer cell line, Ishikawa	1 × 10^−9^ M	MT1 receptor expressing but not MT2 Attenuating ERα mRNA expression Enhancing anti-tumor effects of paclitaxel among anticancer drugs tested	[237]
**Cardiovascular Protection**				
In vivo	Patients with confirmed nocturnal hypertension	2 mg 2 h before bedtime for 4 weeks	Reducing nocturnal systolic and diastolic BP significantly (*p* = 0.01)	[249]
In vivo	Spinal cord injury (SCI) mice model	5, 10, 25, 50, 100 mg/kg i.p.	50 mg/kg exhibiting significantly reduced blood spinal cord barrier permeability Restraining microvessel loss and attenuating edema Protecting the tight junction proteins, endothelial cells and pericytes Decreasing cell apoptosis and reducing MP3/AQP4/HIF-1α/VEGF/VEGFR2 expression	[251]
In vivo	Wistar-Kyoto (WKY) and spontaneously hypertensive rats (SHR)	30 mg/kg/day for 4 weeks	Decreasing reflex chronotropic responses to phenylephrine and sodium nitroprusside Reducing mean arterial pressure and heart rate Improving bradycardic and tachycardic baroreflex responses without modifying catecholamine responses Increasing glutathione peroxidase activity in plasma and erythrocytes	[252]
In vivo	Wistar-Kyoto (WKY) and spontaneously hypertensive rats (SHR)	30 mg/kg/day for 4 weeks	Decreasing mean arterial pressure (MAP) and heart rate Restoring the plasma noradrenaline concentrations, the chronotropic response to isoproterenol and the proportions of β1/β2-adrenoceptors in the heart in SHRs to the levels Decreasing the release of [3H] noradrenaline from isolated atria Improving the relaxation in the aorta	[253]
In vivo	Rats	50 mg/kg	Preventing vasculitis Decreasing elementary pathological lesions of radiation-induced heart disease (RIHD) like fibrosis and necrosis	[254]
In vitro	BM-MSCs	200, 20, and 2 µM(dose-dependent)	Reducing BM-MSC apoptotic death while increasing the levels of TGF-β, bFGF, VEGF, PDGF and Bcl-2, and decreasing Bax, p53 Upregulating modulator of apoptosis (PUMA) and caspase 3 Upregulating the phosphorylation of AMPK, which promotes ACC phosphorylation	[255]
In vivo & in vitro	Female C57BL/6a mice with MI Adipose-derived MSCs	20 mg/kg/day for 28 days 5 µM	Promoting functional survival of AD-MSCs in infarcted heart and provoking a synergetic effect with AD-MSCs to restore heart function associated with alleviated inflammation, apoptosis, and oxidative stress in infarcted heart Exerting cytoprotective effects against hypoxia/serum deprivation (H/SD) injury Attenuating inflammation, apoptosis, and oxidative stress Enhancing SIRT1 signaling, with the increased expression of anti-apoptotic protein Bcl-2, and decreased the expression of Ac-FoxO1, Ac-p53, Ac-NF-KappaB, and Bax.	[256]
In vitro	Perfused isolated rat hearts and cultured neonatal rat cardiomyocytes	5 µM	Improving postischemic cardiac function, decreasing infarct size, reducing apoptotic index, and diminishing lactate dehydrogenase release Upregulating the anti-apoptotic protein Bcl-2 and downregulating Bax Preserving mitochondrial redox potential and elevating SOD activity Decreasing formation of mitochondrial H_2_O_2_ and MDA	[257]
In vivo	Rats with sepsis	30 mg/kg	Improving survival rates and cardiac function, attenuating myocardial injury and apoptosis Decreasing the serum LDH, decreasing inflammatory cytokines TNF-α, IL-1β, and HMGB1 Increasing anti-oxidant enzyme activity and p-Akt and Bcl-2 levels	[258]
In vivo	Drosophila melanogaster	5 µM	Increasing the regularity of heartbeat, rescuing rhythmicity in flies bearing mutations, increasing cardiac regularity independent of alteration of heart rate, which is mediated via a specific G-Protein-coupled receptor encoded by the CG 4313 gene	[259]
In vivo	Patients with heart failure	N/A 1-year follow-up	As a predictors of left ventricular reverse remodeling (LVRR) and the adverse clinical events, increasing the area under of curve for the prediction LVRR	[260]
In vivo	Mice with Mst1 transgenic (Mst1 Tg) and Mst1 knockout (Mst1^−/−^ )	20 mg/kg/d for 1 week	Alleviating postinfarction cardiac remodeling and dysfunction by upregulating autophagy, decreasing apoptosis, and modulating mitochondrial integrity and biogenesis via Mst1/Sirt1 signaling	[261]
**Anti-diabetic Activities**				
In vivo	Albino Wistar rats	10 mg/kg b.w.	Increasing the inhibited activity of catalase in liver cells Restoring the dysfunctional mitochondria related to diabetes	[274]
In vivo	Rat	2.8, 14, 28, and 140 nM	Inhibiting hepatic gluconeogenesis Activating hypothalamic Akt via membrane receptors MT1 and MT2	[275]
In vivo	Rat	10 mg/kg/day	Increasing Ca^2+^ levels in lots of organs and tissues	[11]
In vitro & in vivo	H9C2 cell line Rat	0.1, 1, 10, 100, 1000 µM 20 mg/kg/day	Activating of SIRT1 signaling pathway (significant at 100 and 1000 µM) Inactivating PERK/eIF2α/ATF4 signaling pathway	[276]
In vitro	INS 832/13 cells	1–100 nM	Attenuating β-cell apoptosis, improving β-cell function, prolonging β-cell survival (particularly evident at 10 nM)	[277]
In vivo	Rat	10 mg/kg/day	Improving neurogenesis, synaptogenesis in hippocampi, increasing the receptors of melatonin and insulin, and restoring the downstream signaling pathway for insulin	[278]
In vivo	Rat	250 µg/animal/day/i.p.	Accelerating bone healing	[279]
In vivo	Rat	10 mg/kg/d, i.p.	Restoring the endothelial dysfunction and improving vascular responses	[271]
**Anti-obese Activities**				
In vivo	Rat	10 mg/kg/day	Inducing white adipose tissue browning in rats with obesity-related type 2 diabetes	[282]
In vivo	Rat	20 mg/L	Benefiting homeostasis of renal glutathione	[283]
In vitro	Mouse Gsk3b knockout (Gsk3b^−/−^) and wild-type (Gsk3b^+/+^) MEF cells	10 nM	Inhibiting Akt activation Increasing GSK3B activity	[169]
In vivo	Mice	100 mg/kg/day	Ameliorating obesity-induced adipokine alteration	[285]
In vivo	Women	N/A	Melatonin was involved in the development of obesity	[288]
In vitro	Mice	20 mg/kg/day	Promoting circadian rhythm-mediated proliferation in adipose tissue	[206]
In vivo	Rat	4 mg/kg/day	Decreasing myocardial infarct sizes and insulin resistant Increasing serum PKB/Akt, ERK42/44, GSK-3β and STAT3	[289]
In vivo	Mice	100 mg/kg/day	Increasing mitofusin-2 expression	[290]
In vivo	Mice	10 mg/kg/day	Modulating the MAPK-JNK/p38 signaling pathway	[291]
**Neuroprotection**				
In vivo	Mice	10 mg/kg	Increasing the activity of antioxidant enzymes Mediating the Nrf2-ARE pathway	[293]
In vivo	C57BL/6J mice	10 mg/kg given twice	Reducing IR-induced mitochondrial dysfunction Activating SIRT1 signaling	[294]
In vivo	Rat	150 mg/kg	Suppressing cortical expressions of proinflammatory cytokines	[295]
In vivo	Mice	5 mg/kg	Reducing oxidative damage by scavenging radicals	[296]
In vivo	Rat	10 mg/kg	Reversing the increased plasma TNF-α, IL-1β levels Decreasing BDNF, S100B and IL-10 values	[297]
In vivo	Rat	10 mg/kg and 50 mg/kg	Preventing the decrease of the number and the diameter of sciatic nerve axons	[298]
In vivo	Rat	20 mg	Preventing the decrease in VEPs and PLR Inhibiting microglial reactivity, astrocytosis, demyelination, and axon and retinal ganglion cell loss Preserving anterograde transport of cholera toxin β-subunit	[299]
In vivo	Mice	10 mg/kg	Restoring mRNA and protein levels of BACE1 and PS1	[305]
In vitro & in vivo	Rat hippocampal neurons Rat	50 μM 500 mg/kg b.w.	Improving the soluble Abeta1–42-induced impairment of spatial learning and memory, synaptic plasticity and astrogliosis	[301]
In vivo	Rat	10 mg/kg	Improving motor activity and muscular strength	[306]
In vitro	Mouse neuroblastoma cells	1 μM	Activating transcription factor EB-dependent autophagy-lysosome	[136]
In vivo	Rat	100 mg/kg	Inhibiting caspase-3	[307]
In vivo	Rat	50 mg/kg/day	Protecting the cell against neuronal damage in the hippocampus	[308]
**Other Bioactivities**				
In vivo	Rat	10 mg/kg/day	Improving the microstructure and biomechanical properties of aged bones	[311]
In vivo	Patients	10 mg/day, 60 mg/day	Reducing the hyperoxidative and inflammatory process	[150]
In vivo	Mice	30 mg/kg/day	Decreasing plasma creatine kinase activity, increasing total glutathione content Lowering the oxidized/reduced glutathione ratio	[312]
In vitro	NCI-H292 cells	50, 100, 200, and 400 μM (dose-dependent)	Inhibiting mucin 5AC production	[313]
In vivo	Rat	4 mg/kg, i.p 10 mg/kg, i.p	Exhibits renoprotective effects against ischemia reperfusion induced AKI due to antioxidant properties and the involvement of progesterone receptors	[314]
In vivo	Rat	10 mg/kg/day	Scavenging free radicals	[315]
In vivo	Rat	N/A	Activating SIRT1 signaling	[316]
In vitro	Human ASCs	100 μM for 3 h	Enhancing human ASCs’ survival and their therapeutic effectiveness on injured tissue	[317]
In vivo	Rat	10 mg/body	Interacting with other hormones	[319]
In vivo	Rat	10 mg/kg/day	Decreasing the increased myeloperoxidase activities and osteoclast and neutrophil densities	[322]
In vivo	Rat	10 mg/kg/day	Decreasing serum cyclophosphamide levels and increasing ALP levels	[323]

## Abbreviations

The following abbreviations are used in this manuscript:

2-OHM	2-hydroxymelatonin
4-OHM	4-hydroxymelatonin
6-OHM	6-hydroxymelatonin
AAAD	aromatic L-amino acid decarboxylase
Aβ	amyloid-beta
ACC	acetyl-CoA carboxylase
AD	Alzheimer’s disease
AD-MSCs	adipose-derived mesenchymal stem cells
AFMK	*N*^1^-acetyl-*N*^2^-formyl-5-methoxykynuramine
AMK	*N*^1^-acetyl-5-methoxykynuramine
AMPK	adenosine monophosphate-activated protein kinase
AP-2β	activator protein 2β
ARE	antioxidant responsive element
ATF4	activating transcription factor 4
BACE1	β-APP-cleaving enzyme 1
Bax	Bcl-2-associated X protein
BBB	blood-brain barrier
Bcl-2	B-cell lymphoma 2
BDNF	brain derived neurotrophic factor
bFGF	basic fibroblast growth factor
BMMNCs	bone marrow mononuclear cells
BM-MSCs	bone marrow mesenchymal stem cells
C3-OHM	cyclic 3-hydroxymelatonin
CaMKIIα	calmodulin dependent protein kinase II alpha
cAMP	cyclic AMP
CAT	catalase
CCL20	chemokine C-C motif ligand 20
cGMP	cyclic GMP
CNS	central nerve system
COX-2	cyclo-oxygenase 2
CVDs	cardiovascular diseases
CXCL1	chemokine C-X-C motif ligand 1
cyt c	cytochrome c
DAG	diacylglycerol
DS	diclofenac sodium
DW	dry weight
EBPβ	enhancer-binding protein beta
eIF2α	eukaryotic initiation factor 2α
EMF	electromagnetic fields
EMT	epithelial mesenchymal transition
eNOS	endothelial nitric oxide synthase
ER	endoplasmic reticulum
ERK	extracellular signal-regulated kinase
ERR-α	estrogen-related receptor alpha
FRAP	ferric reducing antioxidant power
FSS	flow shear stress
FW	fresh weight
G6PD	glucose-6-phosphate dehydrogenase
GAS	gamma-activated sequence
γ-GCS	gamma-glutamylcysteine synthetase
G-CSF	granulocyte colony-stimulating factor
GFAP	glia and fibrillary acidic protein
GM-CSF	granulocyte–macrophage colony-stimulating factor
GPx	glutathione peroxidase
GSH-Rd	glutathione reductase
GSK3β	glycogen synthase kinase 3β
H2-Ab1	histocompatibility class II antigen A, beta 1
HDAC	histone deacetylase
Hes1	hairy and enhancer of split 1
HIOMT	hydroxyindole *O*-methyltransferase
HOMA-IR index	homeostasis model assessment of insulin resistance index
hTERT	human telomerase reserve transcriptase
i.v.	intravenously
i.p.	intraperitoneally
IFN-γ	interferon gamma
IGF-1	insulin-like growth factor 1
IKK	inhibitor of nuclear factor kappa-B kinase
IL-6	interleukin 6
iNOS	inducible nitric oxide synthase
IRI	ischemia/reperfusion injury
JAK2	Janus kinase 2
JNK	Jun *N*-terminal kinase
LAN	light at night
LPS	lipopolysaccharide
MAPK	mitogen-activated protein kinase
MDH	malondialdehyde
MI	myocardial infarction
MLHb	medial lateral habenula
MMP-9	matrix metalloproteinase-9
Mst1	mammalian Ste20-like kinase 1
MT1, MT2	melatonin receptors
NAD	Nicotinamide adenine dinucleotide
NAT	*N*-acetyltransferase
NDRG2	*N*-myc downstream-regulated gene 2
NF-κB	nuclear factor kappa-light-chain-enhancer of activated B cells
NK	natural killer
NLRP3	NOD-like receptor P3
NO	nitric oxide
NOMela	*N*-nitrosomelatonin
Nrf2	nuclear factor-erythroid 2-related factor 2
OA	osteoarthritis
ON	optic neuritis
ORAC	oxygen radical antioxidant capacity
Oxa	oxaliplatin
p-Akt	phosphorylated protein kinase B
PARP	poly-ADP-ribose polymerase
pCREB	phosphorylated cAMP response element-binding protein
PD	Parkinson’s disease
PDGF	platelet-derived growth factor
PGC-1α	peroxisome proliferator-activated receptor gamma coactivator-1 alpha
PI3K	phosphatidyl inositol 3-kinase
PKB	protein kinase B
PLR	pupil light reflex
PPAR-γ	peroxisome proliferator-activated receptor gamma
PRDX1	peroxiredoxin 1
PS1	presenilin 1
PUMA	p53 upregulated modulator of apoptosis
RNS	reactive nitrogen species
ROCK-1	Rho-associated kinase protein
ROS	reactive oxygen species
S100B	S100 calcium-binding protein B
SAH	subarachnoid hemorrhage
SCN	suprachiasmatic nucleus
SE	standard error
SEEMs	selective estrogen enzyme modulators
SIRT1	sirtuin 1
SOD	superoxide dismutase
STAT	signal transducer and activator of transcription
TBI	traumatic brain injury
TGF-β	transforming growth factor β
TIMP-1	tissue inhibitor of metalloproteinases 1
TNF-α	tumor necrosis factor α
TPH	tryptophan hydroxylase
UCP1	uncoupling protein 1
UPS	ubiquitin/proteasome system
VEGF	vascular endothelial growth factor
VEP	visual evoked potentials
